# Transparent Topological Meta‐Diplexer via Tightly Confined Valley Surface States

**DOI:** 10.1002/advs.77208

**Published:** 2026-09-03

**Authors:** Sijie Li, Ping Li, Zhihao Lan, Yin Li, Hang Wong, Menglin L.N. Chen

**Affiliations:** ^1^ School of Integrated Circuits Shanghai Jiao Tong University Shanghai China; ^2^ State Key Laboratory of Radio Frequency Heterogeneous Integration, School of Integrated Circuits Shenzhen University Shenzhen China; ^3^ School of Electronic Science and Engineering University of Electronic Science and Technology of China Chengdu China; ^4^ College of Physical Sciences and Engineering Mohammed VI Polytechnic University Ben Guerir Morocco; ^5^ Department of Broadband Communication Peng Cheng Laboratory Shenzhen China; ^6^ State Key Laboratory of Terahertz and Millimeter Waves City University of Hong Kong Hong Kong China

**Keywords:** meta‐diplexer, optical transparency, robust communication, topological metasurface, valley surface states

## Abstract

Topological photonics offers a powerful platform for robust wave manipulation. However, conventional topological devices rely on opaque substrates, limiting their use in applications requiring optical transparency, such as smart windows and transparent electronics. Here, we demonstrate tightly confined valley surface states (VSSs) in an optically transparent topological metasurface platform based on a hollow‐snowflake‐wire design. The proposed platform simultaneously achieves over 85% visible transparency, deep‐subwavelength vertical confinement, and a footprint half that of conventional topological designs. The resulting VSSs exhibit strong robustness against structural discontinuities and high routing flexibility. By tailoring the edge unit cells, we realize a compact topological meta‐diplexer with spatially and spectrally separated channels. Communication experiments under 64‐QAM modulation confirm robust in‐band transmission with error vector magnitude (EVM) <2% and strong inter‐channel isolation with EVM >15% for undesired channels. This work establishes an ultrathin transparent topological metasurface platform, paving the way toward integrated and multifunctional topological photonic systems in optically transparent media.

## Graphical abstract

An optically transparent topological metasurface supporting tightly confined valley surface states enables robust and flexible wave routing in an ultrathin platform. A compact transparent meta‐diplexer with spatially and spectrally separated channels is realized, while communication experiments verify high‐fidelity signal transmission, opening new opportunities for multifunctional transparent topological photonic systems.

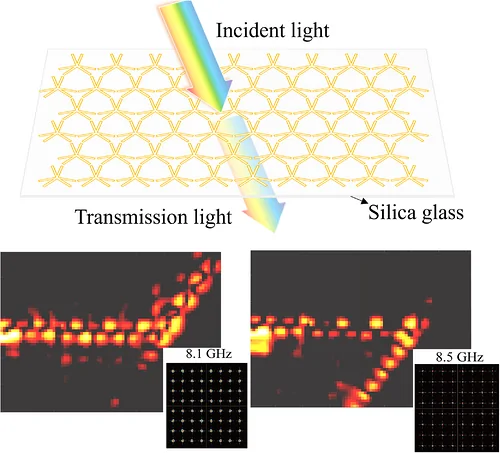

## 1 Introduction

Topological photonics, inspired by the discovery of topological states of matter in condensed matter physics, has emerged as a powerful platform for manipulating electromagnetic waves robustly and unconventionally [1, 2, 3, 4, 5, 6]. Among various topological photonic states, valley interface states (VISs) have been extensively investigated in diverse platforms, including all‐dielectric valley photonic crystals, metal‐based designer surface plasmon crystals (DSPCs), and other topological structures [7]. These valley‐Hall systems offer unique opportunities for robust waveguiding [8, 9, 10], routing [11, 12, 13], and multiplexing [14, 15, 16, 17], owing to their topological protection against backscattering at sharp corners and structural defects.

Beyond different classes of topological platforms, recent efforts have sought to integrate emerging advanced materials into topological photonics to enable new functionalities. Representative approaches include incorporating transparent conducting oxides for ultrafast all‐optical switching [18], exploiting intrinsic topological insulators such as bismuth telluride to enable compact and efficient photonic functionalities [19], and employing two‐dimensional materials like hexagonal boron nitride to extend topological operation toward the visible regime [20]. Besides, transparent materials represent a particularly important class among these emerging materials. These include intrinsically transparent conductors such as indium tin oxide (ITO) and other transparent conductive oxides (TCOs), as well as visually transparent metal structures realized by ultrafine wires or sparse metallic patterns whose feature sizes are below the resolution limit of the human eye. Such transparent materials have been explored to realize optically transparent microwave devices, including transmission lines and metadevices [21, 22, 23, 24, 25, 26]. This transparency is highly desirable for emerging applications requiring optical transparency, such as deployment on vehicle windows, building glass, and integration with photovoltaic elements. However, these transparent platforms generally lack topological protection and therefore suffer from significant scattering loss at defects and bends. Meanwhile, existing topological photonic platforms, although robust against defects, all rely on optically opaque substrates. Consequently, the integration of transparency with topological protection remains an unexplored direction in current devices.

Among various topological platforms, the DSPC architecture is particularly attractive for realizing optical transparency. Its planar geometry offers greater design flexibility for transparent applications compared to all‐dielectric or three‐dimensional topological structures [27, 28]. Typical DSPC‐based VISs are constructed by patterning metal patches on dielectric substrates, where the mirror‐symmetry‐breaking mechanism is exploited to create a valley‐Hall photonic topological insulator [29, 30]. In such designs, strong vertical field confinement is achieved by employing high‐permittivity or thick substrates. Reducing the substrate thickness or permittivity to improve optical transparency would expose the guided waves to air, causing severe radiation loss. As a result, the vertical dimension cannot be scaled down without compromising field confinement, and the required thick or high‐ε substrates are inherently opaque. In addition, conventional VISs typically exhibit large lateral footprints, as they require an interface between two distinct photonic crystals and a sufficiently large size to prevent wave leakage [31, 32, 33, 34]. Recent studies have proposed valley surface states (VSSs) localized at the outer boundary of a single valley‐Hall structure, which reduce the lateral footprint compared to traditional VISs [35, 36, 37]. Nevertheless, their potential for practical device applications remains largely unexplored. Consequently, existing topological DSPC devices remain incompatible with optically transparent applications and still suffer from limited structural compactness.

In this work, we establish an optically transparent topological metasurface platform based on hollow‐snowflake‐shaped metallic wires. By curving the metal lines with deep grooves, we achieve visible transparency larger than 85%, deep‐subwavelength vertical confinement, and a footprint half that of conventional topological designs. We further introduce an equivalent‐circuit framework that directly links structural capacitance and inductance to band dispersions, enabling intuitive tuning of VSSs without full‐wave simulations. The platform supports tightly confined VSSs that preserve topological robustness against sharp bends, while the boundary configuration can be flexibly engineered to achieve a compact footprint and design freedom. Leveraging these features, we experimentally demonstrate the first transparent topological meta‐diplexer supporting 64‐QAM communication, confirming efficient signal transmission and strong inter‐channel isolation.

## 2 Results and Discussion

**FIGURE 1:**
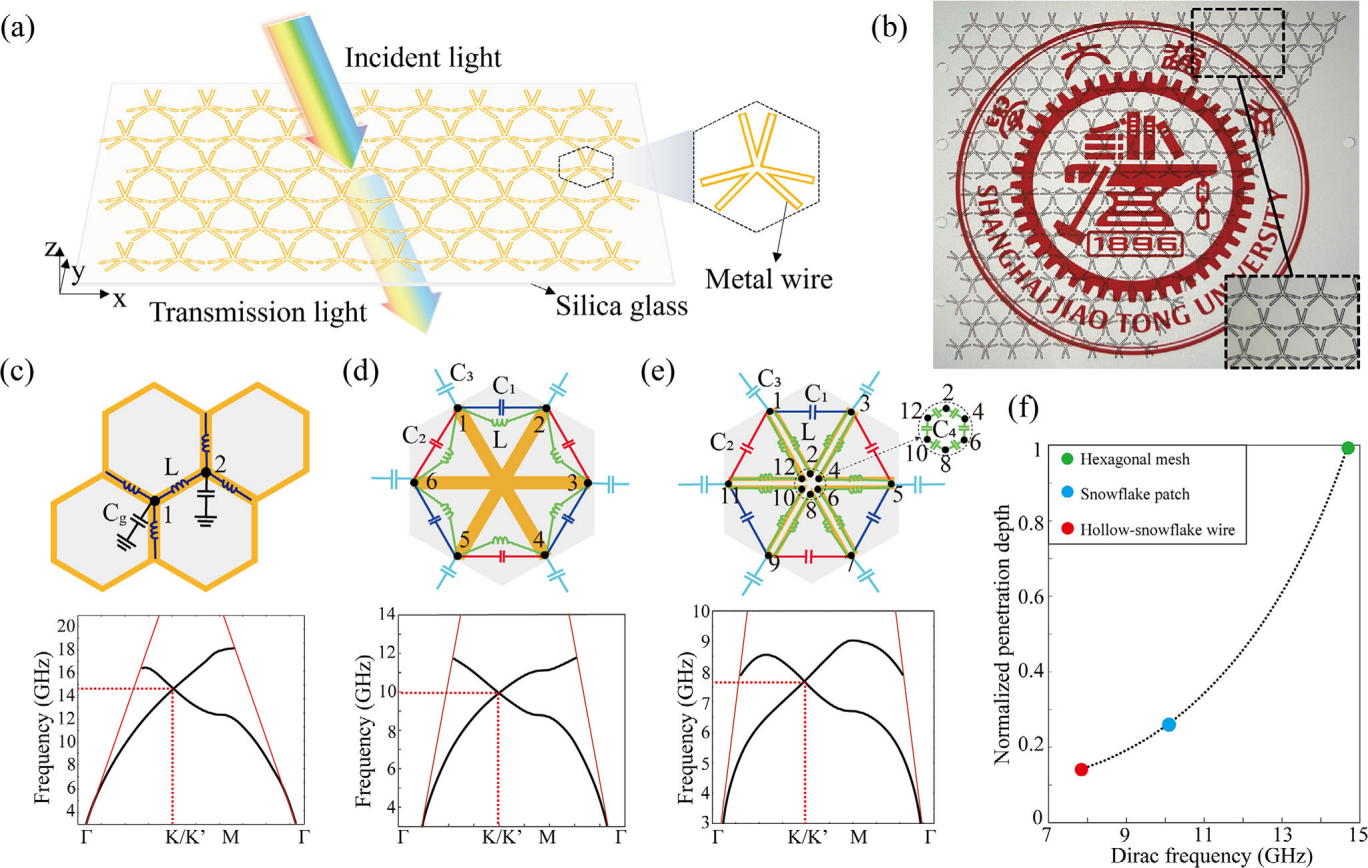
Configuration and characteristics of the hollow‐snowflake‐wire topological metasurface. (a) Schematic of the topological metasurface composed of periodically arranged hollow‐snowflake‐patterned metal wires printed on the silica‐glass substrate. (b) Photograph of the fabricated sample placed above a university emblem pattern, demonstrating its high optical transparency. The structural details are shown in the bottom inset. Metasurface unit cells with $C_{6v}$ symmetry composed of (c) connected hexagonal metal meshes, (d) snowflake‐patterned metal patches, and (e) hollow‐snowflake‐patterned metal wires, together with their corresponding equivalent circuit models and band dispersion diagrams. The hollow‐snowflake‐wire structure has larger equivalent inductance and capacitance so that its Dirac frequency is the lowest. All three structures share the same lattice parameter. The ground‐connected capacitor for each node in structures (d) and (e) are not shown. The solid red lines indicate the light lines. (f) Normalized penetration depth (defined by the $\frac{1}{e}$ decay of the electric field amplitude) versus eigenfrequency for the Dirac‐point eigenstate supported by the above three structures, indicating that a lower Dirac frequency corresponds to a higher vertical confinement and thus a thinner substrate.

Our topological metasurface is composed of periodically arranged hexagonal unit cells of subwavelength size, created from hollow‐snowflake‐patterned metal wires printed on a silica‐glass substrate, as shown in Figure 1a. The metal wires are ultra‐thin, offering excellent optical transparency (>85% in the visible spectrum), as illustrated in Figure 1b. The supported topological guiding modes are localized at the metal–substrate interface and propagate as SSPP modes, whose transmission efficiency is primarily governed by the vertical field confinement of the metal patterns [21].

To construct a topological unit cell with enhanced vertical field confinement, we start with a conventional structure based on the tight binding model, composed of connected hexagonal metal meshes [38, 39] as shown in Figure 1c. The corresponding band dispersion, presented in the lower panel, exhibits a Dirac cone at the K/K' valleys at 14.7 GHz, arising from the $C_{6v}$ symmetry of the honeycomb lattice. By subsequently deforming the hexagonal meshes into snowflake‐patterned metal patches, the Dirac frequency at the K/K' valleys decreases to 10 GHz, as shown in Figure 1d. The decrease of the Dirac frequency widens the momentum mismatch between the eigenstates and free‐space radiation modes, thereby enhancing vertical confinement and suppressing radiation leakage.

To elucidate the mechanism underlying the eigenfrequency reduction, equivalent circuit models are constructed for both structures. The metal wires are modeled as inductors, while the gaps between adjacent wires are represented by capacitors [40]. Besides, a ground‐connected capacitor is assigned to each node to account for the on‐site energy [38, 41]. The band results calculated from the circuit models agree well with the full‐wave simulation results (see Figures S1–S2). According to circuit resonance theory, the frequency reduction from hexagonal meshes to snowflake patches originates from the introduction of additional in‐plane capacitive coupling [42, 43]. Furthermore, increasing the effective inductance also lowers the operating frequency, as demonstrated in Figure S2.

Based on these two mechanisms, the snowflake‐patch structure is further optimized into the hollow‐snowflake‐patterned wire geometry shown in Figure 1e. In this design, narrowing the metal wires increases the inductance L, while an additional capacitor $C_{4}$ is introduced in the equivalent circuit. Consequently, the first two bands are further lowered, and the Dirac frequency is reduced to 7.8 GHz, leading to a larger momentum mismatch between the guided eigenstates and the free‐space modes, and thus a stronger vertical field confinement. Notably, all three structures share the same lattice parameter, so the lower eigenfrequency also corresponds to a deeper subwavelength unit cell, enabling a more compact footprint.

To quantitatively evaluate the vertical field confinement, the penetration depths (defined as the substrate thickness at which the electric field amplitude decays to $\frac{1}{e}$) of the Dirac‐point eigenstates are extracted, as shown in Figure 1f. The normalized penetration depth decreases rapidly with decreasing Dirac frequency, indicating enhanced vertical confinement in metasurfaces with lower Dirac frequencies, in agreement with the band‐dispersion analysis. Notably, the hollow‐snowflake‐wire structure reduces the effective thickness to only 14% of that of a conventional hexagonal‐mesh counterpart, enabling an ultrathin configuration. The corresponding electric field amplitude distributions along the *z*‐direction are provided in Section S2. Consequently, the hollow‐snowflake‐wire structure simultaneously achieves high optical transparency, strong vertical confinement, and a deep‐subwavelength footprint, making it highly advantageous for the miniaturization, integration, and crosstalk suppression of microwave devices. The relationship between optical transparency and electromagnetic performance of the proposed structure is analyzed in Section S3.

**FIGURE 2:**
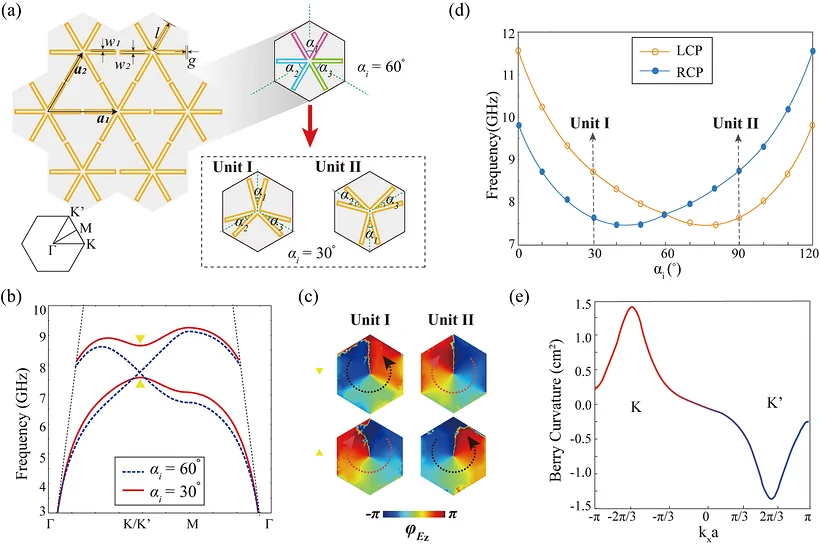
Schematics of the hollow‐snowflake‐wire metasurface and its topological phases. (a) The metasurface consists of hollow‐snowflake‐patterned metal wires. The translation vectors are $a_{1}$ and $a_{2}$ with magnitude a. The wire width, length, inter‐wire gap, and edge spacing are $w_{1}=0.025a$, l=0.425a, $w_{2}=0.025a$ and g=0.025a, respectively. Each branch pair contains two adjacent branches with intersection angle $\alpha_{i}$ (i=1,2,3) and an internal bisector (green dashed lines). By varying the intersection angles from $\alpha_{i}=60^{\circ }$ (i=1,2,3) to $\alpha_{i}=30^{\circ }$ while keeping the internal bisectors fixed, Unit I and its mirror‐symmetric‐counterpart, Unit II, both with $C_{3v}$ symmetry, are constructed. (b) Band dispersions of the first two bands for the $C_{6v}$ ($\alpha_{i}=60^{\circ }$) and $C_{3v}$ ($\alpha_{i}=30^{\circ }$) unit cells. The black dashed lines denote the light lines. (c) Chiralities (dashed arrows) of the phase distributions of the non‐degenerate eigenstates (marked by yellow triangles) in the two topological unit cells. The black arrows indicate left circular polarization (LCP), and the red arrows indicate right circular polarization (RCP). (d) Calculated eigenfrequencies of the LCP and RCP states at the K valley as the parameter $\alpha_{i}$ (i=1,2,3) varies continuously while the internal bisectors are kept fixed. The cases with $\alpha_{i}=60^{\circ }∼120^{\circ }$(i=1,2,3) correspond to the mirror‐symmetric counterparts of the structures with $\alpha_{i}=60^{\circ }∼0^{\circ }$(i=1,2,3). (e) Calculated Berry curvature of the first band for Unit I along the line $k_{y}=2\pi /\left(\sqrt{3}a\right)$ in the momentum space.

The designed hollow‐snowflake‐wire metasurface is depicted in detail in Figure 2a. It consists of hexagonal unit cells arranged in a triangular lattice with lattice constants $\left|\vec{a_{1}}\right|=\left|\vec{a_{2}}\right|=a$. The width and length of metal wires are denoted by $w_{1}$ and l, respectively, while the inter‐wire gap and edge spacing are denoted by $w_{2}$ and g. The substrate has a thickness of 0.125a, a relative permittivity of 3.82, and a loss tangent of $5.7\times 10^{-5}$. Each unit cell comprises six uniformly distributed metal‐wire branches, grouped into three pairs (marked by different colors). Each pair consists of two adjacent branches with an intersection angle $\alpha_{i}$ (i=1,2,3) and an internal bisector, as indicated by the green dashed lines. By fixing the three internal bisectors and rotating the six branches from $\alpha_{i}=60^{\circ }$ (i=1,2,3), corresponding to the $C_{6v}$ symmetric configuration, to $\alpha_{i}=30^{\circ }$ (i=1,2,3), the symmetry is reduced to $C_{3v}$, forming Unit I. Applying mirror symmetry to Unit I with respect to the ΓK direction yields another $C_{3v}$‐symmetric unit cell, denoted as Unit II.

The reduction in symmetry breaks the mirror symmetry of the $C_{6v}$ unit cell along the ΓK direction and lifts the Dirac degeneracy at the K/K' points, thereby opening a complete bandgap from 7.7 to 8.7 GHz, as indicated in Figure 2b. Since time‐reversal symmetry is preserved, the two $C_{3v}$ unit cells share identical band structures. At the K valley, the two non‐degenerate eigenstates exhibit opposite phase vortices, corresponding to left‐ and right‐circular polarizations (LCP and RCP), as shown in Figure 2c. The states at the K' valley are obtained via time‐reversal symmetry, which reverses phase circulation and consequently the circular polarization [44, 45]. Furthermore, by continuously varying $\alpha_{i}$ (i=1,2,3), the eigenfrequencies of the LCP and RCP states at the K valley can be tuned accordingly, as illustrated in Figure 2d (see Section S4 for details). The configurations with $\alpha_{i}=60^{\circ }∼120^{\circ }$ are the mirror‐symmetric counterparts of those with $\alpha_{i}=60^{\circ }∼0^{\circ }$. Notably, the frequency ordering of the LCP and RCP states is inverted as $\alpha_{i}$ crosses $60^{\circ }$, signaling a topological phase transition, consistent with the inversion of phase vortices between Unit I and Unit II shown in Figure 2c.

To characterize the topological phase transition from Unit I to Unit II, we construct an effective Hamiltonian in the vicinity of K/K' valleys using the k·p method [46, 47]

(1) $$H_{K/K^{\prime }}\left(\delta k\right)=\pm v_{D}\delta k_{x}\sigma_{x}+v_{D}\delta k_{y}\sigma_{y}+v_{D}\Delta_{p}\sigma_{z}$$

where $v_{D}$ is the group velocity, $\sigma_{i}\left(i=x,y,z\right)$ are the Pauli matrices, and Δp is proportional to the bandgap width between the first and second bands. Based on the eigenvectors of this Hamiltonian, the local Berry curvature of the first band at the K/K' valleys is given by

(2) $$\Omega_{z,K/K^{\prime }}\left(\delta k\right)=\pm \frac{\Delta_{p}}{2{\left(\delta k^{2}+\Delta_{p}^{2}\right)}^{3/2}}$$

Using Equation(2), we calculate the Berry curvature of Unit I along the line $k_{y}=2\pi /\sqrt{3}a$ in momentum space, as shown in Figure 2e. The opposite Berry curvatures at the K and K' valleys reflect time‐reversal symmetry and the contrasting topological characteristics associated with the two valleys. Moreover, Unit I and Unit II exhibit opposite Berry curvatures at the same valley, confirming that they belong to distinct topological phases.

The valley Chern number serves as a key topological invariant to characterize the valley‐Hall phase. It can be obtained by integrating the local Berry curvature over the half Brillouin zone (HBZ):

(3) $$C^{K/K^{\prime }}=\frac{1}{2\pi }\int_{\mathrm{HBZ}}\Omega_{K/K^{\prime }}\left(\delta k\right)dS=\pm \frac{1}{2}\mathrm{sgn}\left(\Delta_{p}\right)$$

According to the opposite Berry curvature shown in Figure 2e, the above two unit cells possess opposite effective masses and therefore opposite valley Chern numbers. Based on the bulk‐boundary correspondence [48], a topological interface state is expected to emerge when these two configurations are brought into contact.

**FIGURE 3:**
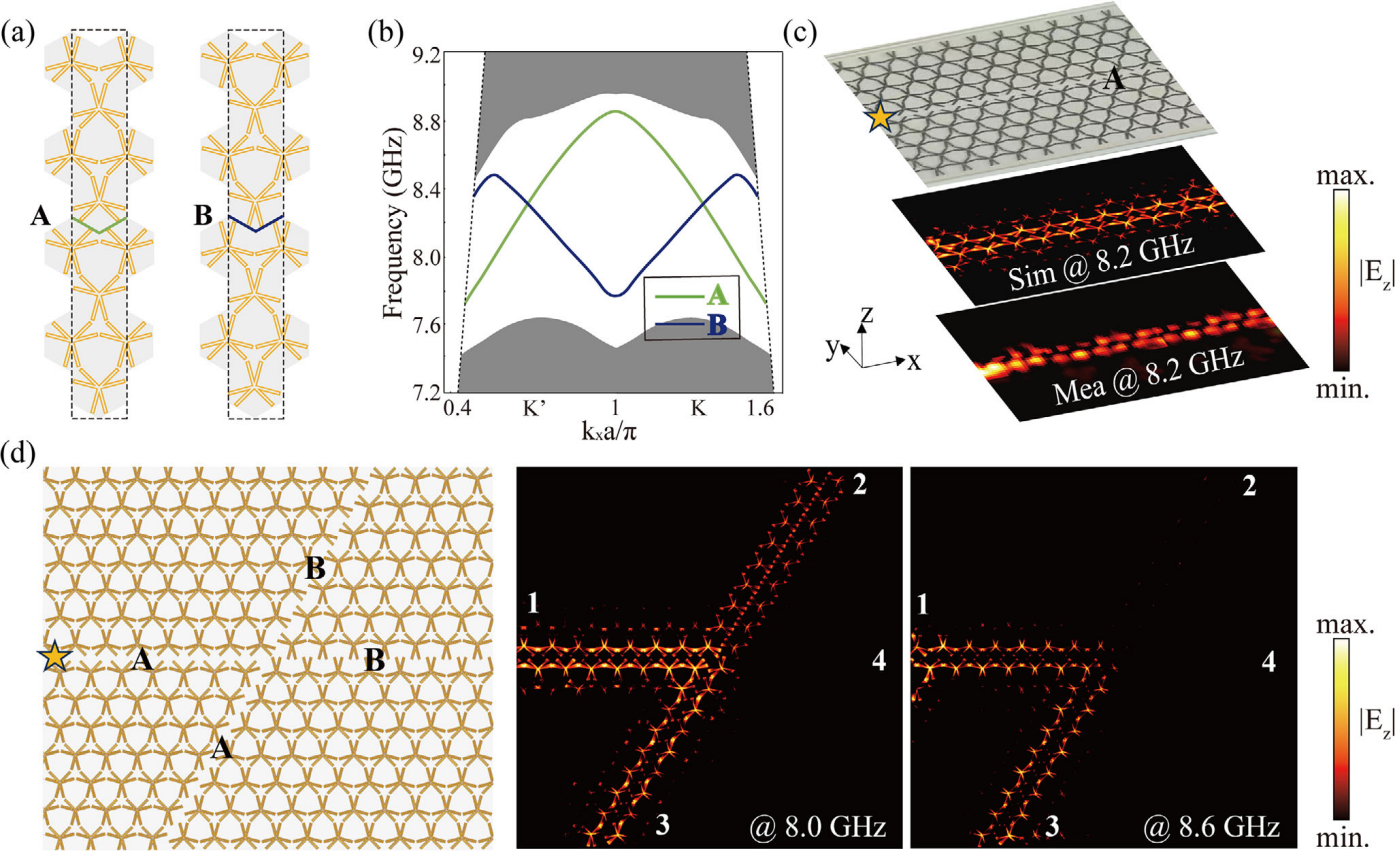
Valley‐polarized interface states supported by topological metasurface and verification of their topological characteristics. (a) TDSPCs A and B are composed of $C_{3v}$ unit cells (Unit I and Unit II) with inverted spatial arrangements. The green and blue lines indicate the interface domain walls, and the dashed rectangles denote the corresponding supercells. (b) Calculated band diagrams of the valley interface states supported by TDSPCs A and B, respectively. The shaded regions represent the projected bulk bands. (c) Fabricated straight waveguide composed of TDSPC A, together with the simulated and measured electric field distributions of the VIS at 8.2 GHz. The yellow star indicates the excitation source. (d) Schematic of the frequency‐dependent multi‐functional device with four interconnected channels formed by TDSPCs A and B. Electric field maps demonstrate a power‐splitting effect within the common frequency bands of VISs A and B, and a routing effect within the frequency band of VIS A only. The maps verify the robust transmission and valley‐momentum locking of the VISs.

As shown in Figure 3a, two topological designer surface plasmon crystals (TDSPCs), denoted as A and B, are constructed by inverting the spatial arrangement of the two valley domains formed by Unit I and Unit II, respectively. Their band diagrams are calculated and presented in Figure 3b. Interface‐state bands appear within the opened bandgap and exhibit valley‐dependent propagation directions, in agreement with the theoretical analysis of the valley Chern numbers [33, 49]:

(4) $$\Delta C_{A}^{K\left(K^{\prime }\right)}=C_{\mathrm{unit}1}^{K\left(K^{\prime }\right)}-C_{\mathrm{unit}2}^{K\left(K^{\prime }\right)}=-1\left(1\right)$$

(5) $$\Delta C_{B}^{K\left(K^{\prime }\right)}=C_{\mathrm{unit}2}^{K\left(K^{\prime }\right)}-C_{\mathrm{unit}1}^{K\left(K^{\prime }\right)}=1\left(-1\right)$$

A straight waveguide constructed by TDSPC A is illustrated in Figure 3c. The VIS is excited by a monopole antenna at the left termination (indicated by the yellow pentagram). Both simulation and experimental results show that the VIS A is tightly confined to the interface domain wall with negligible transverse leakage. To further verify the propagation robustness and valley‐momentum locking of the VIS, a frequency‐dependent multifunctional device is designed, consisting of four interconnected waveguide channels formed by TDSPCs A and B, as shown in Figure 3d. Full‐wave simulations demonstrate that the device functions as a power splitter in the frequency range of 7.8–8.5 GHz, and as a router in the range of 8.5–8.8 GHz, well consistent with the band structure in Figure 3b. In both operating regimes, no power is observed at port 4, confirming the suppression of inter‐valley scattering. Detailed momentum analysis and spatial Fourier spectra are provided in Section S5. In addition, the field map at 8.6 GHz demonstrates that VIS A can propagate robustly around sharp bends under the protection of the bulk topological phases.

The VISs are localized at the interface domain walls between two phase‐inverted valley domains, each requiring at least four rows of unit cells in the transverse direction to maintain lattice periodicity and thus preserve the bulk topological phase. Consequently, the VIS fields occupy only a limited portion of the overall structure, leading to inefficient space utilization and relatively bulky devices. To overcome this limitation, VSSs localized at the external boundaries of valley domains are proposed.

**FIGURE 4:**
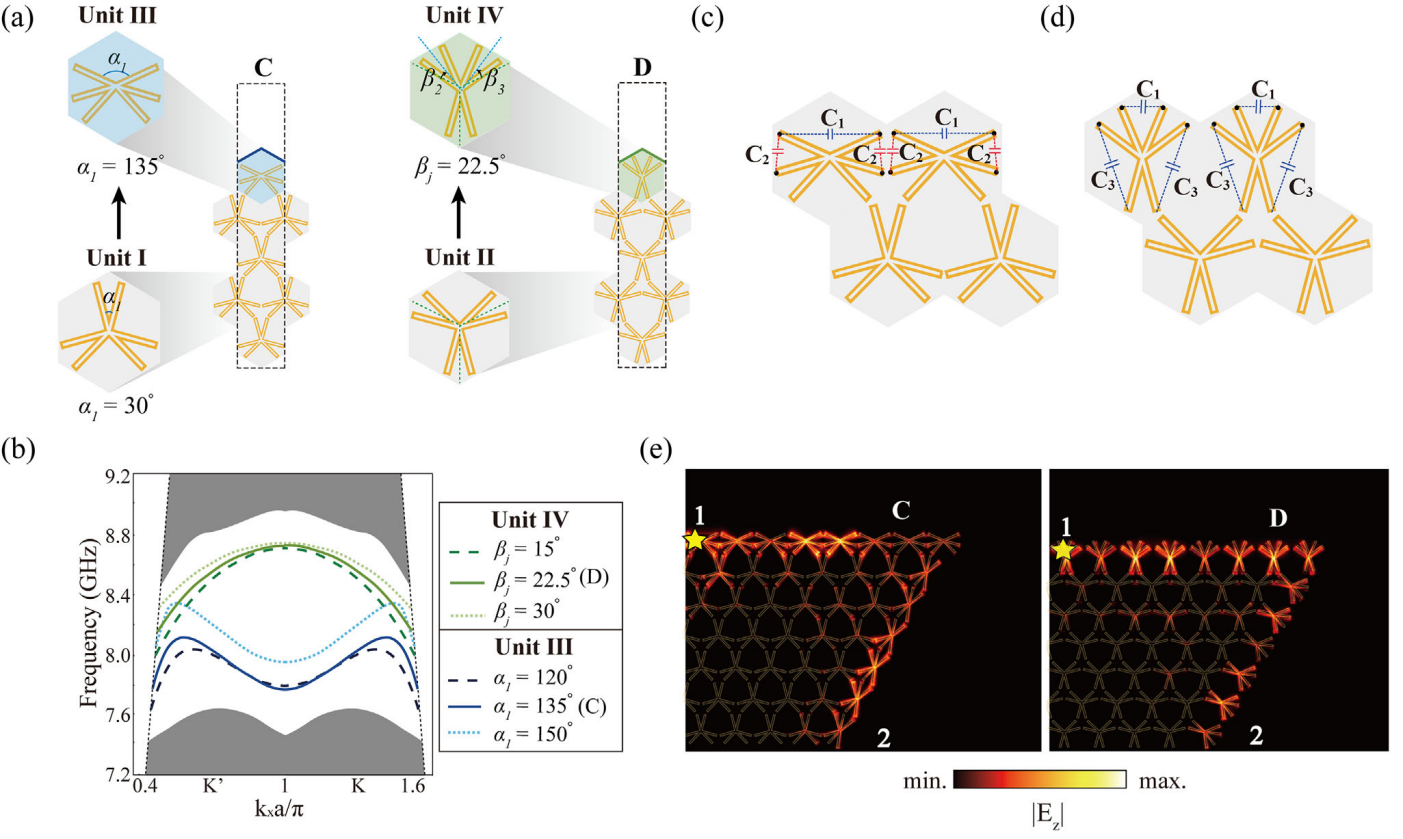
Tunable dispersions of valley‐polarized surface states and verification of their robustness. (a) TDSPC C is constructed with a valley bulk composed of Unit I and replacing the boundary unit cells with the modified Unit III (highlighted in blue). Unit III is formed by rotating the metal branches in Unit I from $\alpha_{1}=30^{\circ }$ to $135^{\circ }$, while keeping the other parameters unchanged. TDSPC D is constructed with a valley bulk composed of Unit II and replacing the boundary unit cells with Unit IV (highlighted in green). Unit IV is formed by rotating the metal branches of Unit II such that the internal bisectors rotate by $\beta_{j}=22.5^{\circ }$ (j=2,3), while the other parameters remain unchanged. (b) Band dispersion diagrams of valley surface states with tailorable boundary unit cells. The three blue lines at lower frequencies correspond to the VSS bands of TDSPC C with varying $\alpha_{1}$ in Unit III, while the three green lines at higher frequencies correspond to the VSS bands of TDSPC D with varying $\beta_{j}$ in Unit IV. The solid lines indicate the cases shown in (a). (c) Equivalent‐circuit components varying with $\alpha_{1}$ in TDSPC C. A larger $\alpha_{1}$ corresponds to a larger $C_{2}$ and a smaller $C_{1}$. (d) Equivalent‐circuit components varying with $\beta_{j}$ in TDSPC D. A larger $\beta_{j}$ corresponds to a larger $C_{1}$ and a smaller $C_{3}$, while the other unshown components keep unchanged. (e) Field profiles of surface waveguides composed of TDSPCs C and D with $120^{\circ }$ bends, respectively, showing robust propagation of valley surface states through sharp bends.

As shown in Figure 4a, TDSPC C is constructed with a valley bulk composed of Unit I, while the external boundary unit cells are replaced by Unit III (highlighted in blue). Unit III is built by modifying the intersection angle $\alpha_{1}$ in Unit I from $30^{\circ }$ to $135^{\circ }$, while keeping the other parameters fixed. Similarly, TDSPC D consists of a Unit II‐based bulk and a Unit IV‐based boundary (highlighted in green). Unit IV is built by rotating the metal branches of Unit II to $\beta_{j}=22.5^{\circ }$(j=2,3) while keeping the other parameters unchanged. The VSSs supported at the external boundaries of TDSPCs C and D share the same topological origin as the VISs, both arising from inversion‐symmetry breaking and the associated nontrivial valley topology. Unlike VISs, which are supported by the interface between two topologically distinct valley domains, VSSs are supported by the interplay between the bulk valley topology and a properly terminated external boundary.

The boundary matrix condition $\hat{M}=\tau_{z}\sigma_{y}$ is essential for engineering VSSs [35, 50, 51]. For zigzag terminations, the effective boundary matrix is given by:

(6) $$\begin{array}{c} {\hat{M}}_{\text{zigzag}\;}=\tau_{z}\left(\sigma_{y}\cdot \sin\theta_{V}+\sigma_{z}\cdot \cos\theta_{V}\right), \end{array}$$

where $\tau_{z}$ is the Pauli matrix and $\theta_{V}$ is determined by the on‐site edge potentials. In our structure, the on‐site edge potential is engineered by rotating the metal branches of the boundary unit cells, thereby contributing to the VSS band shifting correspondingly as shown in Figure 4b. Figure 4c,d illustrates the equivalent circuit components that vary with the branch rotation in TDSPCs C and D, respectively. As $\alpha_{1}$ increases in Unit III, capacitor $C_{1}$ decreases while the adjacent capacitors $C_{2}$ increase due to changes in the wire gap width, resulting in an upward shift of the VSS band in TDSPC C, as shown by the three blue lines in Figure 4b. Similarly, as $\beta_{j}$ increases at Unit IV, capacitor $C_{1}$ increases, while capacitors $C_{3}$ decrease, which also shifts the VSS band upward in the dispersion diagram, as shown by the three three lines in Figure 4b. The equivalent circuit results and geometrical details for the four perturbed unit cells are provided in Sections S6 and S7.

Benefiting from the efficient and convenient analysis enabled by the generalized eigenvalue formulation of the equivalent circuit model, VSSs C and D are designed to operate in fully separated frequency ranges of 7.7–8.1 GHz and 8.2–8.7 GHz, respectively. The large momentum mismatch induced by the tailored on‐site edge potentials ensures strong edge confinement of the VSSs, effectively suppressing radiation leakage into free space. To verify their propagation robustness, two waveguides incorporating $120^{\circ }$ sharp bends are constructed using TDSPCs C and D, respectively, as shown in Figure 4e. The field distributions demonstrate that the VSSs, excited at port 1, propagate smoothly around the sharp bends toward port 2 with negligible loss, confirming their valley‐locked propagation and intrinsic topological protection. Moreover, the VSS exhibits excellent propagation robustness against local defects comparable to that of the VIS (in Section S8).

**FIGURE 5:**
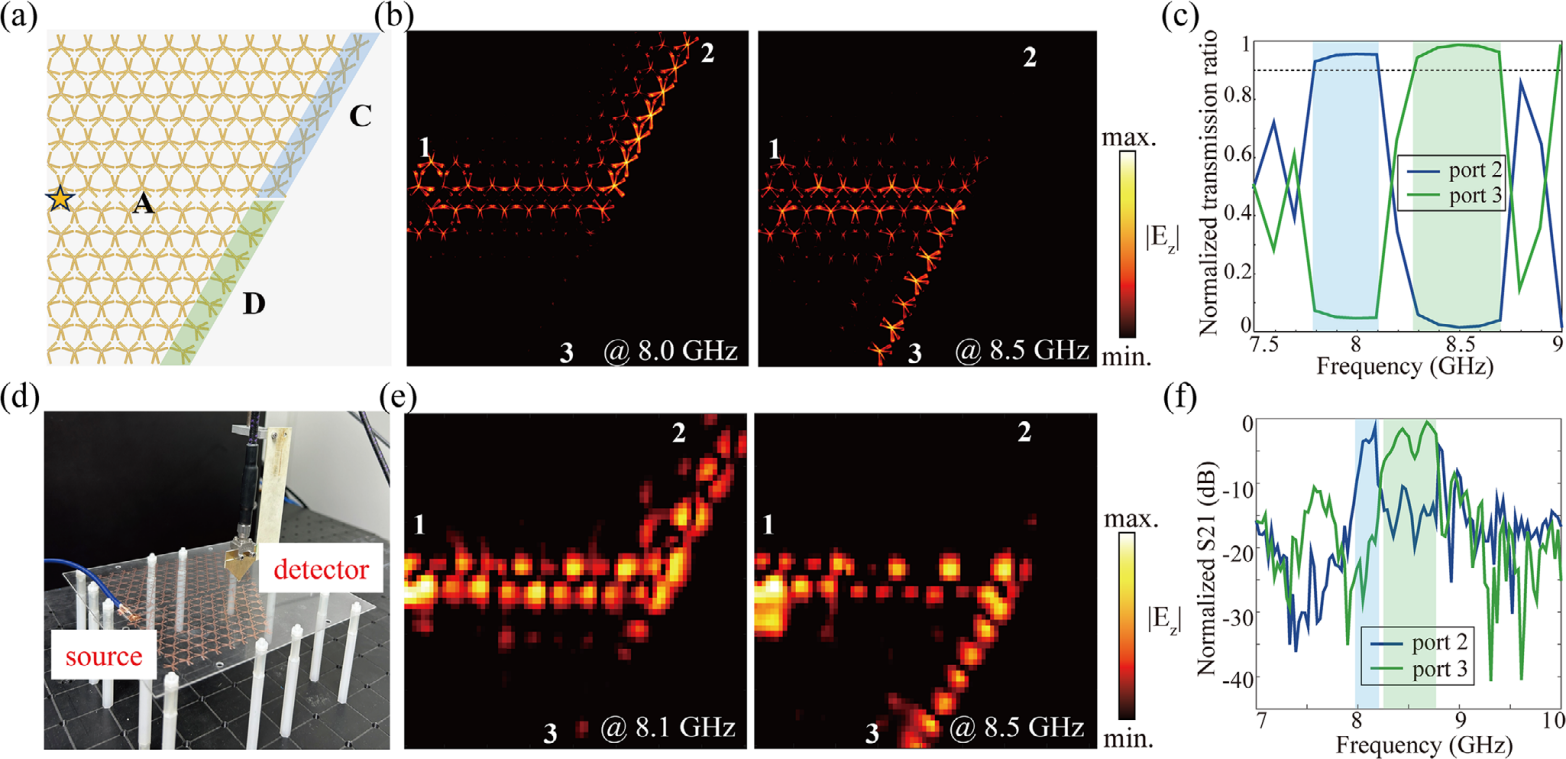
Configuration and operation performance of the topological diplexer. (a) The schematics of the proposed topological diplexer, formed by truncating the straight waveguide based on TDSPC A by $60^{\circ }$, with the upper and lower surface boundaries replaced by TDSPCs C and D, respectively. (b) The simulated electric field maps of the diplexer operating at distinct frequencies. (c) Simulated normalized transmission spectra of the diplexer. The blue and green shaded regions indicate the frequency ranges in which more than 90% of the power is directed to port 2 and port 3, respectively. (d) Photograph of the fabricated sample during near‐field scanning measurement. The source is a monopole antenna, and the $E_{z}$ field detector is mounted on the scanning robotic arm. (e) Measured electric field maps of the diplexer operating at distinct frequencies, showing robust propagation and high channel isolation. (f) Experimentally measured normalized transmission spectra of the diplexer, showing high isolation performance.

Leveraging the VIS supported by TDSPC A and the spectrally separated VSSs supported by TDSPCs C and D, we design a topological meta‐diplexer with over 85% visible transparency. As shown in Figure 5a, the topological diplexer is constructed by truncating the straight waveguide formed by TDSPC A at a $60^{\circ }$ angle, and replacing its upper and lower boundaries with TDSPCs C and D, respectively. Since TDSPCs C and D support VSSs with non‐overlapping frequency ranges, the energy carried by VIS A selectively couples to either VSS C or VSS D depending on the operating frequency, as illustrated by the simulated field distributions in Figure 5b. The mode transition occurs smoothly at the interconnection region with minimal loss. Importantly, all three modes—VIS A, VSS C, and VSS D—share the same valley momentum (see Section S5), ensuring momentum‐matched and reflection‐free coupling. To quantify the demultiplexing performance, the normalized transmission spectra at the two output ports are calculated and plotted in Figure 5c. Over 90% of the input power is directed to port 2 within 7.8–8.1 GHz, and to port 3 within 8.3–8.7 GHz, well consistent with the VSS bandwidths presented above.

To experimentally characterize the meta‐diplexer, a physical sample is fabricated by patterning an 8‐um‐thick copper layer on a 1‐mm‐thick silica‐glass substrate using photolithography. The near‐field scanning measurement setup is shown in Figure 5d. A monopole antenna source and an $E_{z}$ field detector are connected to port 1 and port 2 of a vector network analyzer (VNA), respectively, to measure the transmission parameter S21, which is proportional to the $E_{z}$ component. The detector is positioned 1 mm above the sample and scans in the *xy*‐plane (see Section S9 for details). The measured field distributions are plotted in Figure 5e, directly visualizing the frequency‐dependent directional routing behavior of the topological diplexer. The normalized measured S21 spectra are shown in Figure 5f, indicating that the topological states are routed to port2 in the range of 7.9–8.2 GHz and to port3 in the range of 8.3–8.7 GHz. The slight frequency deviations from the simulations are attributed to fabrication tolerances (in Section S10) and measurement uncertainties. Both simulation and measurement results demonstrate that the proposed topological diplexer achieves robust and frequency‐dependent routing. In addition, a unidirectionally‐excited topological router and another topological meta‐diplexer are presented in Sections S11 and S12, respectively, further demonstrating the flexible routing capability and strong inter‐channel isolation enabled by the VSSs.

**FIGURE 6:**
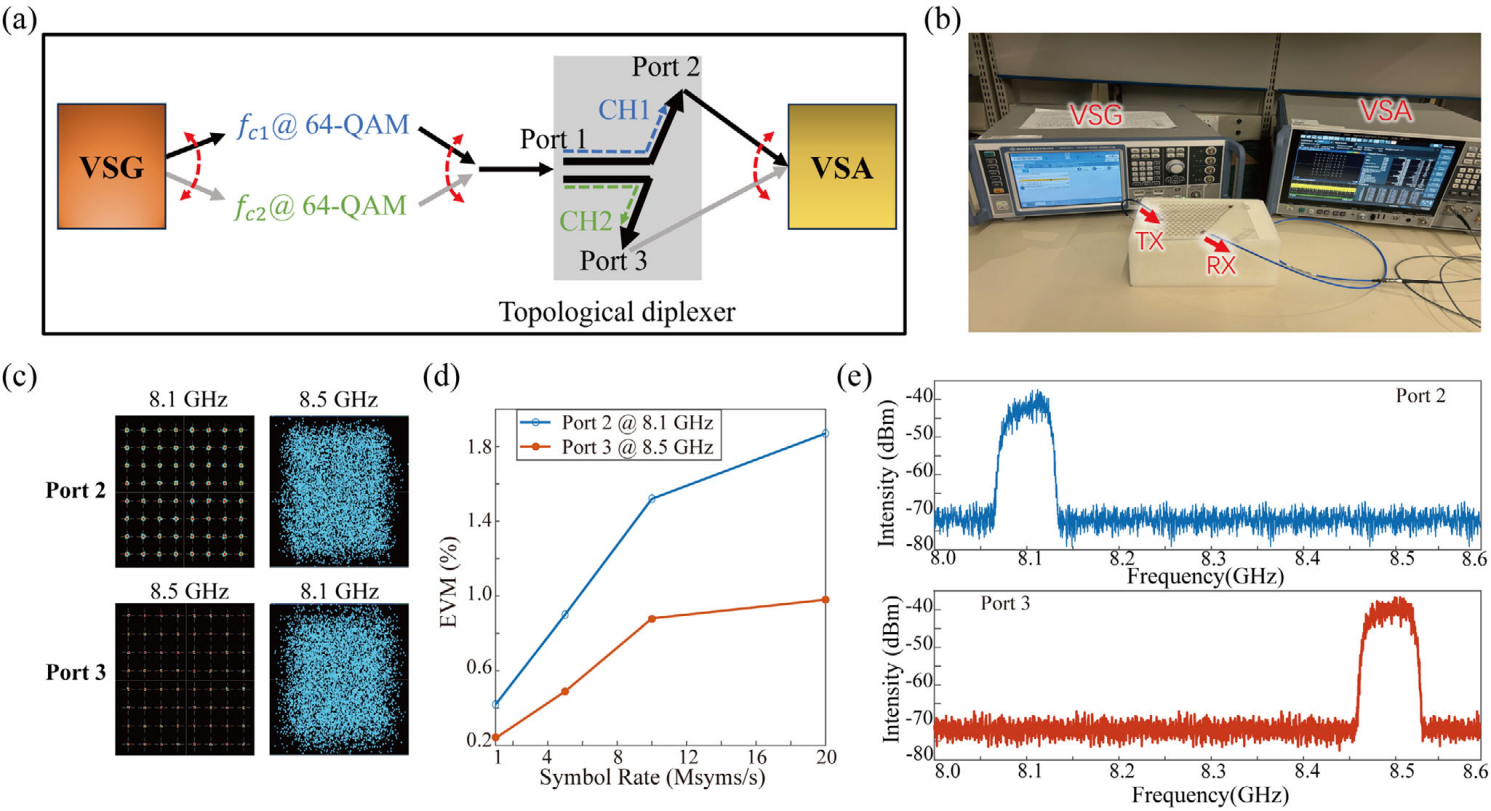
Communication experiment setup and measurement results of the topological diplexer. (a) Schematic of the communication measurement setup used to characterize the topological diplexer with 64‐QAM signals at carrier frequencies $f_{c1}$ and $f_{c2}$. (b) Image shows the fabricated sample in the communication experiment. The diplexer is placed on foam with a dielectric constant close to 1. (c) IQ constellation diagrams of the demodulated 64‐QAM signals with a symbol rate of 10 Msyms/s at Port 2 and Port 3 for $f_{c1}=8.1$ GHz and $f_{c2}=8.5$ GHz, respectively. (d) EVM as a function of symbol rate for both output ports at their corresponding central frequencies. (e) Measured absolute spectral power of the 50 Msyms/s input signals in the two channels centered at carrier frequencies of 8.1 and 8.5 GHz.

Furthermore, to quantitatively evaluate the signal transmission and demultiplexing performance of the topological meta‐diplexer in a communication system, a communication experiment is conducted, as shown in Figure 6a. A vector signal generator (VSG) is used to generate 64‐QAM signals centered at carrier frequencies $f_{c1}$(8.1 GHz) or $f_{c2}$(8.5 GHz). The transmitted signals are coupled into Port 1 of the diplexer via a monopole antenna and received at Port 2 or Port 3 through another monopole antenna. The received signals are subsequently demodulated and analyzed using a vector signal analyzer (VSA). A photograph of the experimental setup is shown in Figure 6b, where the diplexer is placed on a foam substrate with a dielectric constant near to 1. Figure 6c presents the in‐phase and quadrature (IQ) constellation diagrams of the received signals at Ports 2 and 3 for both carrier frequencies at a symbol rate of 10 Msyms/s. Additional IQ diagrams for different symbol rates are provided in Section S13. It is observed that channel 1 effectively transmits signals centered at 8.1 GHz with low distortion while strongly suppresses signals at 8.5 GHz, whereas channel 2 exhibits the opposite behavior. These results confirm low‐loss guided‐wave transmission and high inter‐channel isolation in the topological meta‐diplexer.

To further quantify the transmission quality, error vector magnitude (EVM) analysis is performed at Ports 2 and 3 for their respective passband signals. As shown in Figure 6d, the EVM increases with symbol rate but remains below 2%, indicating excellent signal integrity and near‐lossless transmission. The slightly lower signal quality at Port 2 compared to Port 3 is attributed to the broader field distribution in TDSPC C than in TDSPC D, as shown in Figure 5b,e. Since signals are received by a monopolar antenna, less power is coupled out at Port 2, resulting in marginally degraded performance. Inter‐channel interference is further evaluated, yielding an EVM above 15% in the undesired channels, indicating strong interference suppression. Figure 6e shows the measured absolute spectral power of the 50 Msyms/s signals centered at 8.1 and 8.5 GHz at Port 2 and Port 3, respectively, which is used to evaluate the in‐band transmission flatness. Moreover, a short data‐stream transmission test is performed, yielding a bit error rate (BER) of zero on 4,80,000 transmitted bits, as depicted in Section S14. These results demonstrate that the proposed topological meta‐diplexer achieves excellent transmission quality and strong channel isolation, making it a competitive component for practical communication systems.

Both near‐field scanning and communication experiments confirm that the proposed topological diplexer achieves efficient transmission and strong channel isolation. A quantitative comparison with representative reported topological multiplexers is provided in Section S15, demonstrating that the proposed diplexer features minimum structural size and unprecedented optical transparency. Benefiting from the compact footprint and ultrathin profile enabled by the tailorable VSS and the hollow‐snowflake‐wire architecture, the proposed optically transparent topological platform offers significant potential for flexible routing and device miniaturization, paving the way for multifunctional integrated topological metasurface devices.

## 3 Conclusion

In this work, we develop a transparent topological metasurface platform composed of hollow‐snowflake‐patterned metal wire printed on a silica‐glass substrate, which supports topological states with enhanced field confinement and reduces the substrate thickness to only 14% of a conventional hexagonal‐mesh counterpart. An equivalent circuit model with efficient computational capability is employed to guide the structural design and elucidate the tunability of the surface states, enabling the proposed design to be readily scaled to other frequency regimes while preserving the underlying valley topological mechanism. Both valley interface states and surface states exhibit valley‐momentum conservation and robust propagation against structural discontinuities. In particular, the designed valley surface states enable strong inter‐channel isolation and flexible routing capability. By exploiting the seamless transition between interface and surface states, a compact topological meta‐diplexer is numerically designed and experimentally demonstrated. Communication experiments further verify robust signal transmission and excellent channel isolation under 64‐QAM modulation. This work establishes a multifunctional topological metasurface platform that combines optical transparency with ultrathin configuration, paving the way for integrated topological photonic systems.

## 4 Experimental Section

### 4.1 Numerical Simulation

All band diagrams of the topological unit cells and edge states were calculated utilizing the finite‐element method in COMSOL Multiphysics. Mesh convergence study is conducted for the thin metal‐wire geometry to confirm the numerical accuracy in Section S16. The boundary conditions are set with Floquet periodic boundary or scattering boundary condition according to the structures. The propagation results of the structures are also verified by CST Microwave Studio time‐domain solvers. All equivalent circuit calculations are conducted with MATLAB.

### 4.2 Fabrication of Sample

The samples were fabricated using standard photolithography. An 8‐um‐thick copper layer was first deposited onto a 1‐mm‐thick silica glass substrate by vacuum evaporation. Subsequently, a photoresist layer was spin‐coated onto the copper surface and selectively exposed to patterned ultraviolet light. After exposure, the exposed regions of the positive photoresist were removed, defining the desired pattern. The unprotected copper was then etched using a wet chemical process, transferring the pattern to the copper layer. Finally, the remaining photoresist was stripped, and the samples were cleaned and thermally treated to remove residual contaminants.

### 4.3 Measurement Setups

For near‐field measurement, a stripped coaxial cable, with its outer conductor removed, was used as a monopole antenna and fixed at the left end of the channel to excite the sample. An electric‐field probe sensitive to the out‐of‐plane component Ez was employed as the detector and mounted on a motorized scanning arm. During the measurements, the probe was positioned at a constant height of 1 mm above the sample surface to record the local electric field. Both the excitation antenna and the Ez probe were connected to a vector network analyzer, from which the transmission coefficient S21 was obtained. For the communication measurement, two monopole antennas are used as excitation source and detector and are fixed at Port 1 and Port 2 (3) of the sample, respectively. The vector signal generator is ROHDE& SCHWARZ SMW200A, and transmits modulated signals at a level of 0 dBm. The vector signal analyzer is ROHDE& SCHWARZ FSW signal & spectrum analyzer, demodulating and analyzing the received signals.

## Conflicts of Interest

The authors declare no conflicts of interest.

## Supporting information


**Supporting File**: advs77208‐sup‐0001‐SuppMat.pdf.

## Data Availability

The data that support the findings of this study are available from the corresponding author upon reasonable request.

## References

[1] J. E. Moore , “The Birth of Topological Insulators,” Nature 464, no. 7286 (2010): 194–198. 20220837 10.1038/nature08916

[2] M. Z. Hasan and C. L. Kane , “Colloquium: Topological Insulators,” Reviews of Modern Physics 82 (2010): 3045–3067.

[3] X.‐L. Qi and S.‐C. Zhang , “Topological Insulators and Superconductors,” Reviews of Modern Physics 83 (2011): 1057–1110.

[4] L. Lu , J. D. Joannopoulos , and M. Soljačić , “Topological Photonics,” Nature Photonics 8, no. 11 (2014): 821–829.

[5] Z. Wang , Y. Chong , J. D. Joannopoulos , and M. Soljačić , “Observation of Unidirectional Backscattering‐Immune Topological Electromagnetic States,” Nature 461, no. 7265 (2009): 772–775. 19812669 10.1038/nature08293

[6] Y. Poo , R.‐X. Wu , Z. Lin , Y. Yang , and C. T. Chan , “Experimental Realization of Self‐Guiding Unidirectional Electromagnetic Edge States,” Physical Review Letters 106, no. 9 (2011): 093903. 21405623 10.1103/PhysRevLett.106.093903

[7] Z. Lan , M. L. Chen , F. Gao , S. Zhang , and W. E. Sha , “A Brief Review of Topological Photonics in One, Two, and Three Dimensions,” Reviews in Physics 9 (2022): 100076.

[8] R. Zhou , M. L. N. Chen , X. Shi , et al., “Protected Transverse Electric Waves in Topological Dielectric Waveguides,” IEEE Transactions on Antennas and Propagation 72, no. 2 (2024): 2058–2063.

[9] Z. Gao , F. Gao , Y. Zhang , Y. Luo , and B. Zhang , “Flexible Photonic Topological Insulator,” Advanced Optical Materials 6, no. 17 (2018): 1800532.

[10] R. Zhou , X. Shi , H. Lin , et al., “Super‐Robust Telecommunications Enabled by Topological Half‐Supermodes,” Advanced Science 13, no. 13 (2026): e15157. 41517867 10.1002/advs.202515157 PMC12955864

[11] X. Zhang , S. Li , Z. Lan , W. Gao , and M. L. N. Chen , “Reconfigurable Photonic Valley Filter in Hybrid Topological Heterostructures,” Laser & Photonics Reviews 19, no. 3 (2025): 2400797.

[12] J. W. You , Q. Ma , Z. Lan , Q. Xiao , N. C. Panoiu , and T. J. Cui , “Reprogrammable Plasmonic Topological Insulators with Ultrafast Control,” Nature Communications 12, no. 1 (2021): 5468. 10.1038/s41467-021-25835-6 PMC8443663 34526488

[13] X. Zhang , S. Li , C. Yu , W. Gao , and M. L. N. Chen , “Reconfigurable Topological Power Router Based on Interferometric Edge States,” IEEE Transactions on Antennas and Propagation 74 (2026): 4985–4989.

[14] X.‐T. He , C.‐H. Guo , G.‐J. Tang , M.‐Y. Li , X.‐D. Chen , and J.‐W. Dong , “Topological Polarization Beam Splitter in Dual‐Polarization All‐Dielectric Valley Photonic Crystals,” Physical Review Applied 18, no. 4 (2022): 044080.

[15] A. Kumar , M. Gupta , P. Pitchappa , et al., “Phototunable Chip‐Scale Topological Photonics: 160 Gbps Waveguide and Demultiplexer for THz 6G Communication,” Nature Communications 13, no. 1 (2022): 5404. 10.1038/s41467-022-32909-6 PMC9478161 36109511

[16] J. Ma , C. Ouyang , Y. Yang , et al., “Asymmetric Frequency Multiplexing Topological Devices Based on a Floating Edge Band,” Photonics Research 12, no. 6 (2024): 1201–1212.

[17] M. Gupta , A. Kumar , P. Pitchappa , et al., “150 Gbps THz Chip‐Scale Topological Photonic Diplexer,” Advanced Materials 36, no. 19 (2024): 2309497. 10.1002/adma.202309497 38350050

[18] Z. A. Kudyshev , A. V. Kildishev , A. Boltasseva , and V. M. Shalaev , “Tuning Topology of Photonic Systems with Transparent Conducting Oxides,” ACS Photonics 6, no. 8 (2019): 1922–1930.

[19] D. Singh , S. Nandi , Y. Fleger , S. Z. Cohen , and T. Lewi , “Deep‐Subwavelength Resonant Meta‐Optics Enabled by Ultra‐High Index Topological Insulators,” Laser & Photonics Reviews 17, no. 9 (2023): 2200841.

[20] M. Wu , Y. Yang , H. Fei , et al., “Unidirectional Transmission of Visible Region Topological Edge States in Hexagonal Boron Nitride Valley Photonic Crystals,” Optics Express 30, no. 4 (2022): 6275–6283. 35209568 10.1364/OE.439769

[21] H.‐R. Zu , B. Wu , B. Chen , et al., “Optically and Radiofrequency‐Transparent Metadevices Based on Quasi‐One‐Dimensional Surface Plasmon Polariton Structures,” Nature electronics 6, no. 7 (2023): 525–533.

[22] L. Li , P. Zhang , F. Cheng , M. Chang , and T. J. Cui , “An Optically Transparent Near‐Field Focusing Metasurface,” IEEE Transactions on Microwave Theory and Techniques 69, no. 4 (2021): 2015–2027.

[23] B. Chen , B. Wu , H.‐R. Zu , J.‐Q. Hou , and T. Su , “Experimental Demonstration of High Optically Transparent Reflectarrays Using Fine Metal Line Structure,” IEEE Transactions on Antennas and Propagation 70, no. 11 (2022): 10504–10511.

[24] C. Fan , W. Zhao , K. Chen , J. Zhao , T. Jiang , and Y. Feng , “Optically Transparent Conformal Reflectarray with Multi‐Angle Microwave Efficient Scattering Enhancement,” Advanced Optical Materials 12, no. 18 (2024): 2400151.

[25] P. Aghabeyki , P. de la Rosa , M. Ca no‐Garc'ıa , X. Quintana , R. Guirado , and S. Zhang , “Optically Transparent Beam‐Steering Reflectarray Antennas Based on a Liquid Crystal for Millimeter‐Wave Applications,” IEEE Transactions on Antennas and Propagation 72, no. 1 (2023): 614–627.

[26] W. Li , F. Li , W. Tang , et al., “Stretchable Antenna Arrays‐Enabled Multi‐Target Balloon Wireless Communication System,” Advanced Functional Materials 36 (2025): e10521.

[27] Y. Li , S. Xu , Z. Zhang , et al., “Polarization‐Orthogonal Nondegenerate Plasmonic Higher‐Order Topological States,” Physical Review Letters 130, no. 21 (2023): 213603. 37295078 10.1103/PhysRevLett.130.213603

[28] F. Gao , Z. Gao , X. Shi , et al., “Probing Topological Protection Using a Designer Surface Plasmon Structure,” Nature Communications 7, no. 1 (2016): 11619. 10.1038/ncomms11619 PMC4876474 27197877

[29] X. Wu , Y. Meng , J. Tian , Y. Huang , H. Xiang , D. Han , and W. Wen , “Direct Observation of Valley‐Polarized Topological Edge States in Designer Surface Plasmon Crystals,” Nature Communications 8, no. 1 (2017): 1304. 10.1038/s41467-017-01515-2 PMC5670222 29101323

[30] L. Chen , X. Yu Li , J. L. Su , et al., “Flexible and Robust Metasurface‐Based Wearable Sensor for Intelligent Human Monitoring,” Advanced Materials 38, no. 10 (2026): e14150. 41451579 10.1002/adma.202514150

[31] M. L. N. Chen , L. J. Jiang , Z. Lan , and W. E. I. Sha , “Coexistence of Pseudospin‐ and Valley‐Hall‐Like Edge States in a Photonic Crystal with $C_{3v}$ Symmetry,” Physical Review Research 2 (2020): 043148.

[32] E. Wen , D. J. Bisharat , R. J. Davis , X. Yang , and D. F. Sievenpiper , “Designing Topological Defect Lines Protected by Gauge‐Dependent Symmetry Indicators,” Physical Review Applied 17, no. 6 (2022): 064008.

[33] T. Ma and G. Shvets , “All‐Si Valley‐Hall Photonic Topological Insulator,” New Journal of Physics 18, no. 2 (2016): 025012.

[34] S. Li , M. L. N. Chen , Z. Lan , and P. Li , “Coexistence of Large‐Area Topological Pseudospin and Valley States in a Tri‐Band Heterostructure System,” Optics Letters 48, no. 17 (2023): 4693–4696. 37656588 10.1364/OL.501977

[35] R. Xi , Q. Chen , Q. Yan , et al., “Topological Chiral Edge States in Deep‐Subwavelength Valley Photonic Metamaterials,” Laser & Photonics Reviews 16, no. 11 (2022): 2200194.

[36] J. Han , F. Liang , Y. Zhao , et al., “Valley Kink States and Valley‐Polarized Chiral Edge States in Substrate‐Integrated Topological Photonic Crystals,” Physical Review Applied 21, no. 1 (2024): 014046.

[37] M. L. Chen , L. Jun Jiang , Z. Lan , and W. E. Sha , “Local Orbital‐Angular‐Momentum Dependent Surface States with Topological Protection,” Optics Express 28, no. 10 (2020): 14428–14435. 32403483 10.1364/OE.387993

[38] Y. Li , Y. Sun , W. Zhu , et al., “Topological LC‐Circuits Based on Microstrips and Observation of Electromagnetic Modes with Orbital Angular Momentum,” Nature Communications 9, no. 1 (2018): 4598. 10.1038/s41467-018-07084-2 PMC6214976 30389947

[39] D. J. Bisharat and D. F. Sievenpiper , “Electromagnetic‐Dual Metasurfaces for Topological States Along a 1D Interface,” Laser & Photonics Reviews 13, no. 10 (2019): 1900126.

[40] M. Li , S. Xiao , J. Long , and D. F. Sievenpiper , “Surface Waveguides Supporting Both TM Mode and TE Mode with the Same Phase Velocity,” IEEE Transactions on Antennas and Propagation 64, no. 9 (2016): 3811–3819.

[41] S. Liu , W. Gao , Q. Zhang , et al., “Topologically Protected Edge State in Two‐Dimensional Su–Schrieffer–Heeger Circuit,” Research 2019 (2019): 8609875. 31549092 10.34133/2019/8609875 PMC6750084

[42] J. Y. Yin , J. Ren , H. C. Zhang , Q. Zhang , and T. J. Cui , “Capacitive‐Coupled Series Spoof Surface Plasmon Polaritons,” Scientific Reports 6, no. 1 (2016): 24605. 27089949 10.1038/srep24605 PMC4835726

[43] C. H. Wu , L. Shen , H. Zhang , et al., “Equivalent Circuit Parameters of Planar Transmission Lines with Spoof Surface Plasmon Polaritons and Its Application in High‐Density Circuits,” Scientific Reports 9, no. 1 (2019): 18853. 31827144 10.1038/s41598-019-55200-z PMC6906369

[44] J. Lu , C. Qiu , M. Ke , and Z. Liu , “Valley Vortex States in Sonic Crystals,” Physical Review Letters 116, no. 9 (2016): 093901. 26991176 10.1103/PhysRevLett.116.093901

[45] T. Ma and G. Shvets , “All‐Si Valley‐Hall Photonic Topological Insulator,” New Journal of Physics 18, no. 2 (2016): 025012.

[46] J. Mei , Y. Wu , C. T. Chan , and Z.‐Q. Zhang , “First‐Principles Study of Dirac and Dirac‐Like Cones in Phononic and Photonic Crystals,” Physical Review B—Condensed Matter and Materials Physics 86, no. 3 (2012): 035141.

[47] H. Wang , L. Xu , H. Chen , and J.‐H. Jiang , “Three‐Dimensional Photonic Dirac Points Stabilized by Point Group Symmetry,” Physical Review B 93, no. 23 (2016): 235155.

[48] M. Ezawa , “Topological Kirchhoff Law and Bulk‐Edge Correspondence for Valley Chern and Spin‐Valley Chern Numbers,” Physical Review B 88, no. 16 (2013): 161406.

[49] X.‐D. Chen , F.‐L. Zhao , M. Chen , and J.‐W. Dong , “Valley‐Contrasting Physics in All‐Dielectric Photonic Crystals: Orbital Angular Momentum and Topological Propagation,” Physical Review B 96, no. 2 (2017): 020202.

[50] W. Yao , S. A. Yang , and Q. Niu , “Edge States in Graphene: From Gapped Flat‐Band to Gapless Chiral Modes,” Physical Review Letters 102, no. 9 (2009): 096801. 19392547 10.1103/PhysRevLett.102.096801

[51] X. Xi , J. Ma , S. Wan , C.‐H. Dong , and X. Sun , “Observation of Chiral Edge States in Gapped Nanomechanical Graphene,” Science Advances 7, no. 2 (2021): eabe1398. 33523977 10.1126/sciadv.abe1398 PMC7787500

