# Supplementary material for: Transparent Topological Meta‐Diplexer via Tightly Confined Valley Surface States
Source: Adv Sci (Weinh). 2026 Sep 3:e77208. Online ahead of print. doi: 10.1002/advs.77208 (PMC13542408; doi:10.1002/advs.77208)
Supplement: Supplementary file 1 [file ADVS-9999-e77208-s001.pdf]

Supporting Information for

Transparent Topological Meta-Diplexer via Tightly Confined Valley Surface States

Sijie Li[1,2], Ping Li[3*], Zhihao Lan[4], Yin Li[5], Hang Wong[6], Menglin L.N. Chen[2†]

[1]School of Integrated Circuits, Shanghai Jiao Tong University, Shanghai 200240, China

[2]State Key Laboratory of Radio Frequency Heterogeneous Integration, School of Integrated Circuits, Shenzhen University, Shenzhen 518060, China

[3]School of Electronic Science and Engineering, University of Electronic Science and Technology of China, Chengdu 611730, China

[4]College of Physical Sciences and Engineering, Mohammed VI Polytechnic University, Ben Guerir 43150, Morocco

[5]Department of Broadband Communication, Peng Cheng Laboratory, Shenzhen, China

[6]State Key Laboratory of Terahertz and Millimeter Waves, City University of Hong Kong, Hong Kong, China

Emails: [*]ping.li@uestc.edu.cn; [†]mlchen@szu.edu.cn

S1. Equivalent circuit model calculation of the metasurface unit cells

S2. The electric field distributions along z direction of the three metasurfaces

S3. Relationship between optical transparency and electromagnetic performance

S4. Explanation for the frequency shifting as α_i varying

S5. Spatial Fourier spectra of the topological power splitter and diplexer

S6. Equivalent circuit calculation of the tailorable valley-polarized surface states

S7. The profiles of perturbed boundary unit cells

S8. The quantitative robustness performance against local defects of VIS and VSS

S9. The Ez field probe for near-field measurement

S10. Fabrication tolerances of the transparent DSPC

S11. A frequency-dependent topological router with unidirectional-excitation property

S12. Another topological diplexer configuration

S13. IQ constellation diagrams for different symbol rates

S14. Short data stream communication performance measurement

S15. Quantitative comparison of the topological diplexers/multiplexers

S16. Mesh convergence study of the hollow-snowflake-wire geometry

S1. Equivalent circuit model calculation of the metasurface unit cells

The equivalent circuit model of the connected-hexagonal-mesh structure (in figure 1(c)) is extracted and shown in figure. S1. To calculate the eigenfrequencies of this periodic circuit, we apply the Kirchhoff's current law to each node in the unit cell, the current that flows out of each node as:

$$I_1 = \frac{\left(V_1 - V_2 e^{-i\mathrm{k}\cdot\vec{a}_1}\right)}{i\omega L} + \frac{\left(V_1 - V_2\right)}{i\omega L} + \frac{\left(V_1 - V_2 e^{-i\mathrm{k}\cdot\vec{a}_2}\right)}{i\omega L} + V_1 i\omega C \tag{S1}$$

$$I_2 = \frac{\left(V_2 - V_1\right)}{i\omega L} + \frac{\left(V_2 - V_1 e^{i\vec{k}\cdot\vec{a}_2}\right)}{i\omega L} + \frac{\left(V_2 - V_1 e^{i\vec{k}\cdot\vec{a}_1}\right)}{i\omega L} + V_2 i\omega C \tag{S2}$$

Where $\vec{a}_1 = (a,0)$ and $\vec{a}_2 = (\frac{a}{2},\frac{\sqrt{3}a}{2})$ are the lattice vectors, and $\vec{k} = (k_x,k_y)$ is the momentum space vectors. Writing Eqs. (S1)-(S2) in the following form,

$$\begin{pmatrix} I_1 \\ I_2 \end{pmatrix} = \boldsymbol{J} \begin{pmatrix} V_1 \\ V_2 \end{pmatrix} \tag{S3}$$

where **J** is the grounded Laplacian matrix,

$$\boldsymbol{J} = i\begin{pmatrix} \omega \mathrm{C} - \dfrac{3}{\omega L} & \dfrac{1 + e^{-i\vec{k}\cdot\vec{a}_1} + e^{-i\vec{k}\cdot\vec{a}_2}}{\omega L} \\ \dfrac{1 + e^{i\vec{k}\cdot\vec{a}_1} + e^{i\vec{k}\cdot\vec{a}_2}}{\omega L} & \omega \mathrm{C} - \dfrac{3}{\omega L} \end{pmatrix} \tag{S4}$$

Then, by solving $det[\,\mathbf{J}(\omega,\vec{k})\,] = 0$, we can obtain the eigenvalues of the periodic circuit at different Bloch wave vectors [1], producing the 2D band structure as shown in the figure S1, which remains consistent with the full-wave simulation results.

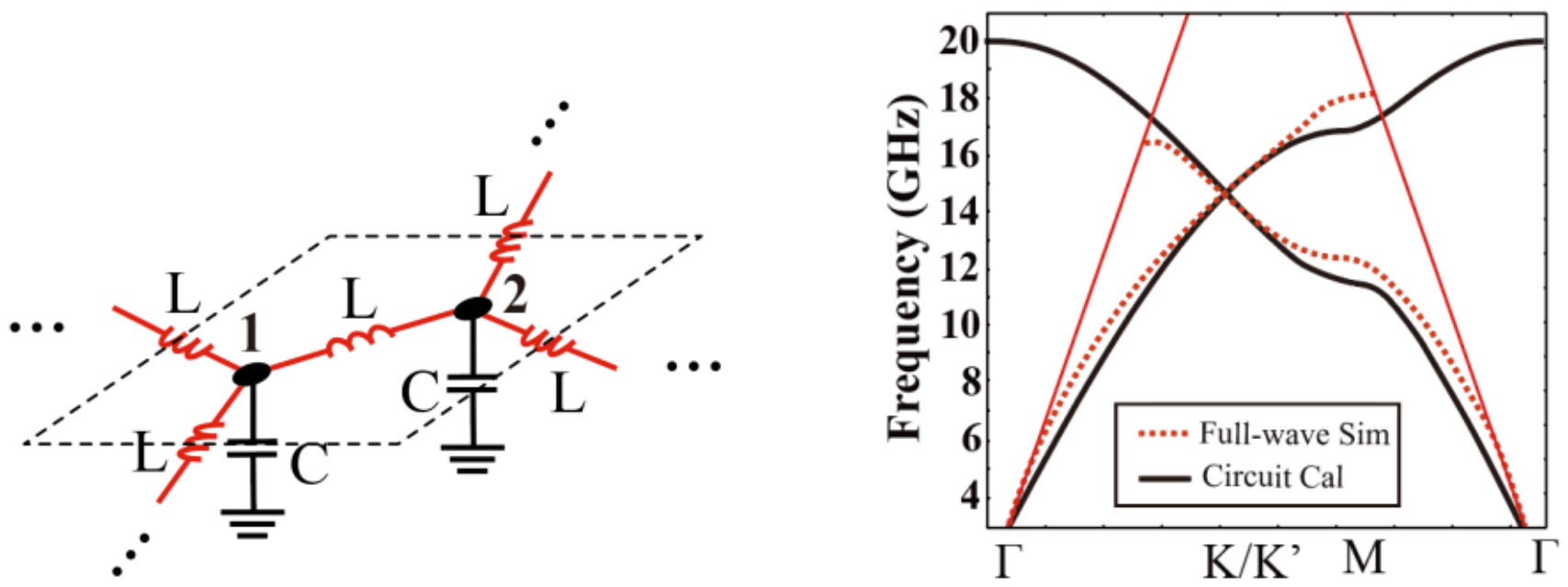

Figure S1. Equivalent circuit model and the calculated bands of the connected hexagonal mesh structure in figure 1(c).

In principle, the aforementioned approach of calculating the circuit eigenvalues to obtain the band structure is applicable to arbitrary periodic circuits. However, as the geometry of the metallic patches becomes more complex, the corresponding equivalent circuit also increases in complexity. A single unit cell may then contain six or even twelve nodes, causing the dimension of the grounded Laplacian matrix to increase from the original 2×2 to 6×6, or even 12×12. Since solving a zero-determinant condition is essentially a nonlinear root-finding problem, the computational complexity increases rapidly with matrix size. The situation becomes even more prohibitive when calculating the band

structure of supercells composed of multiple unit cells, where the matrix dimension grows proportionally with the total number of nodes, making it difficult to obtain the band structures of edge states based on the grounded Laplacian matrix method.

To address this issue, we introduce a **generalized eigenvalue formulation**, which transforms the problem from a complex nonlinear formulation into a linear algebra framework, thereby enabling efficient computation of complex and large-scale circuit models. The equivalent circuit model of snowflake-patch structure (in figure 1(d)) is extracted and shown in figure S2, composing six nodes within each unit cell. Similar with before, we apply the Kirchhoff's current Law to the six nodes, and the current that flows out of each node as:

$$I_1=\frac{(V_1-V_2)}{i\omega L}+\frac{(V_1-V_6)}{i\omega L}+(V_1-V_2)i\omega C_1+(V_1-V_6)i\omega C_1+\left(V_1-V_4e^{i\vec{k}\cdot\vec{a_3}}\right)i\omega C_2+V_1 i\omega C_3 \tag{S5}$$

$$I_2=\frac{(V_2-V_1)}{i\omega L}+\frac{(V_2-V_3)}{i\omega L}+(V_2-V_1)i\omega C_1+(V_2-V_3)i\omega C_1+\left(V_2-V_5e^{i\vec{k}\cdot\vec{a_2}}\right)i\omega C_2+V_2 i\omega C_3 \tag{S6}$$

$$I_3=\frac{(V_3-V_2)}{i\omega L}+\frac{(V_3-V_4)}{i\omega L}+(V_3-V_2)i\omega C_1+(V_3-V_4)i\omega C_1+\left(V_3-V_6e^{i\vec{k}\cdot\vec{a_1}}\right)i\omega C_2+V_3 i\omega C_3 \tag{S7}$$

$$I_4=\frac{(V_4-V_3)}{i\omega L}+\frac{(V_4-V_5)}{i\omega L}+(V_4-V_3)i\omega C_1+(V_4-V_5)i\omega C_1+\left(V_4-V_1e^{-i\vec{k}\cdot\vec{a_3}}\right)i\omega C_2+V_4 i\omega C_3 \tag{S8}$$

$$I_5=\frac{(V_5-V_4)}{i\omega L}+\frac{(V_5-V_6)}{i\omega L}+(V_5-V_4)i\omega C_1+(V_5-V_6)i\omega C_1+\left(V_5-V_2e^{-i\vec{k}\cdot\vec{a_2}}\right)i\omega C_2+V_5 i\omega C_3 \tag{S9}$$

$$I_6=\frac{(V_6-V_5)}{i\omega L}+\frac{(V_6-V_1)}{i\omega L}+(V_6-V_5)i\omega C_1+(V_6-V_1)i\omega C_1+\left(V_6-V_3e^{-i\vec{k}\cdot\vec{a_1}}\right)i\omega C_2+V_6 i\omega C_3 \tag{S10}$$

Where $\vec{a_1}=(a,0)$, $\vec{a_2}=(\frac{a}{2},\frac{\sqrt{3}a}{2})$ and $\vec{a_3}=(-\frac{a}{2},\frac{\sqrt{3}a}{2})$ are the lattice vectors, and $\vec{k}=(k_x,k_y)$ is the momentum space vectors. According to the Kirchhoff's current Law, we can apply $I_i=0(i=1\sim 6)$ to the Eqs. (S5)-(S10). By rearranging all components involving the inductance L to the right side, the equation (S5) can be written as:

$$(V_1-V_2)\omega^2C_1+(V_1-V_6)\omega^2C_1+\left(V_1-V_4e^{i\vec{k}\cdot\vec{a_3}}\right)\omega^2C_2+V_1\omega^2C_3=\frac{(V_1-V_2)}{L}+\frac{(V_1-V_6)}{L} \tag{S11}$$

Eqs. (S6)-(S10) can also be transferred to the similar formula. Then, we can write them in the following form,

$$\omega^2\mathbf{CV}=\mathbf{L}^{-1}\mathbf{V} \tag{S12}$$

Where $\mathbf{C}$ and $\mathbf{L}^{-1}$ are the capacitance and inductance matrix, respectively,

$$\mathbf{C}=\begin{pmatrix} 2C_1+C_2+C_3 & -C_1 & 0 & -C_2e^{i\vec{k}\cdot\vec{a_3}} & 0 & -C_1 \\ -C_1 & 2C_1+C_2+C_3 & -C_1 & 0 & -C_2e^{i\vec{k}\cdot\vec{a_2}} & 0 \\ 0 & -C_1 & 2C_1+C_2+C_3 & -C_1 & 0 & -C_2e^{i\vec{k}\cdot\vec{a_1}} \\ -C_2e^{-i\vec{k}\cdot\vec{a_3}} & 0 & -C_1 & 2C_1+C_2+C_3 & -C_1 & 0 \\ 0 & -C_2e^{-i\vec{k}\cdot\vec{a_2}} & 0 & -C_1 & 2C_1+C_2+C_3 & -C_1 \\ -C_1 & 0 & -C_2e^{-i\vec{k}\cdot\vec{a_1}} & 0 & -C_1 & 2C_1+C_2+C_3 \end{pmatrix} \tag{S13}$$

$$\mathbf{L}^{-1}=\begin{pmatrix} \frac{2}{L} & -\frac{1}{L} & 0 & 0 & 0 & -\frac{1}{L} \\ -\frac{1}{L} & \frac{2}{L} & -\frac{1}{L} & 0 & 0 & 0 \\ 0 & -\frac{1}{L} & \frac{2}{L} & -\frac{1}{L} & 0 & 0 \\ 0 & 0 & -\frac{1}{L} & \frac{2}{L} & -\frac{1}{L} & 0 \\ 0 & 0 & 0 & -\frac{1}{L} & \frac{2}{L} & -\frac{1}{L} \\ -\frac{1}{L} & 0 & 0 & 0 & -\frac{1}{L} & \frac{2}{L} \end{pmatrix} \tag{S14}$$

Then, by solving the generalized eigenvalues of the Eq. (S12) at different Bloch wave vectors, we can obtain the band structure efficiently as shown in the figure S2, which remains consistent with the full-wave simulation results.

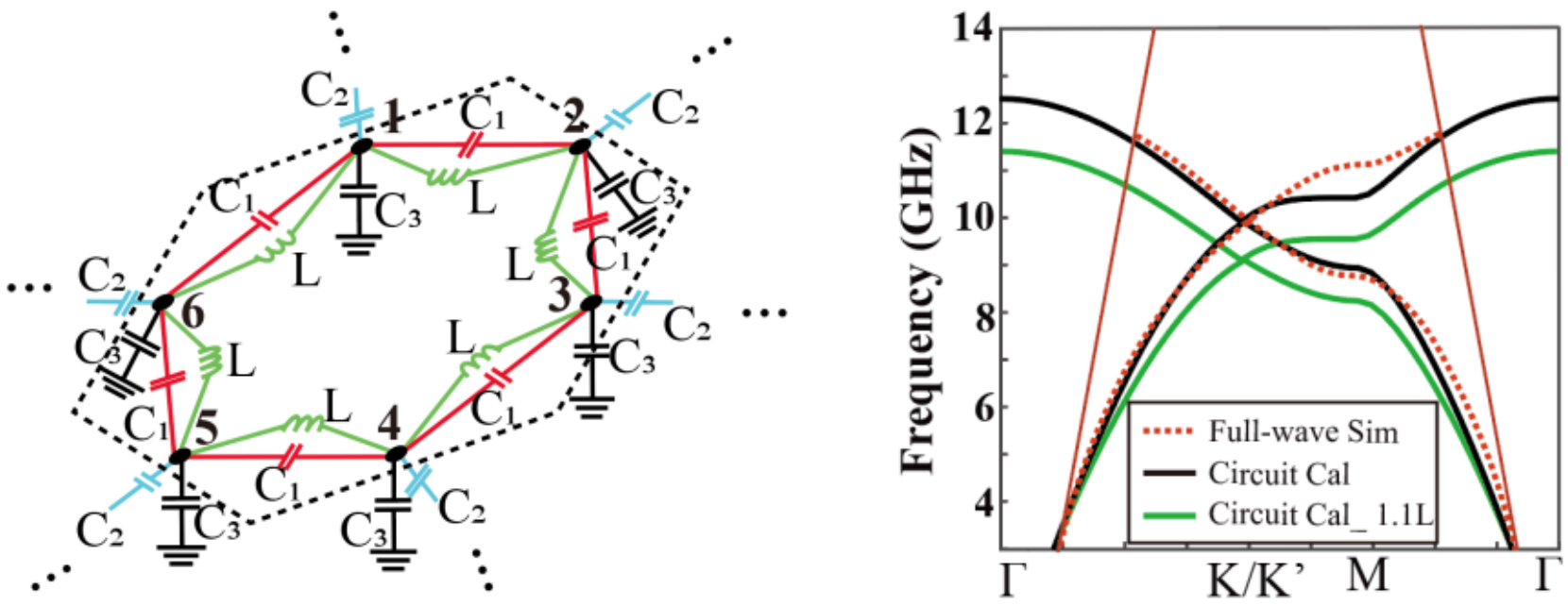

Figure S2. Equivalent circuit model and the calculated bands of the snowflake-patch structure. The green solid lines in the band diagram indicate the calculated bands for increasing L.

The band structure calculation of periodic circuits is reformulated from the conventional approach of solving the characteristic equation $\det(\cdot)=0$ to a generalized eigenvalue problem. While fully preserving the accuracy of the results, this transformation significantly improves computational efficiency, enabling rapid analysis of complex and large-scale circuit structures. As a consequence, the band structures of unitcell, supercell, and bulk circuits can be readily obtained.

Given that the equivalent circuit model enables rapid and efficient calculation of band structures, we tune the circuit element parameters to obtain compressed eigenfrequencies, and then map these parameter variations back to the corresponding physical structure. By keeping all other circuit parameters unchanged and simultaneously increasing L by a factor of 1.1, the overall band shape remains unchanged, while the frequencies are uniformly shifted downward, as indicated by the green solid lines shown in figure S2. This result demonstrates that increasing the equivalent inductance in

the equivalent circuit leads to a reduction in operating frequency, consistent to the circuit resonance theory. In the physical metasurface structure, an increase in the equivalent inductance corresponds to a decrease in the width of the metal patch branches. Since the effect achievable by reducing w alone is limited, the patch geometry is further optimized into a hollow-snowflake-wire configuration, as shown in Figure 1(e).

The equivalent circuit model of the hollow-snowflake-wire structure is extracted and shown in figure S3. Same to the snowflake-patch structure, generalized eigenvalue formulation is applied, where the capacitance and inductance matrix possess 12×12 sizes because of 12 nodes contained in a unit cell. The calculated bands are calculated as indicated in figure S3, generally consistent with the simulated bands. The slight discrepancy arises because certain next-nearest-neighbor coupling terms cannot be fully captured by the equivalent model.

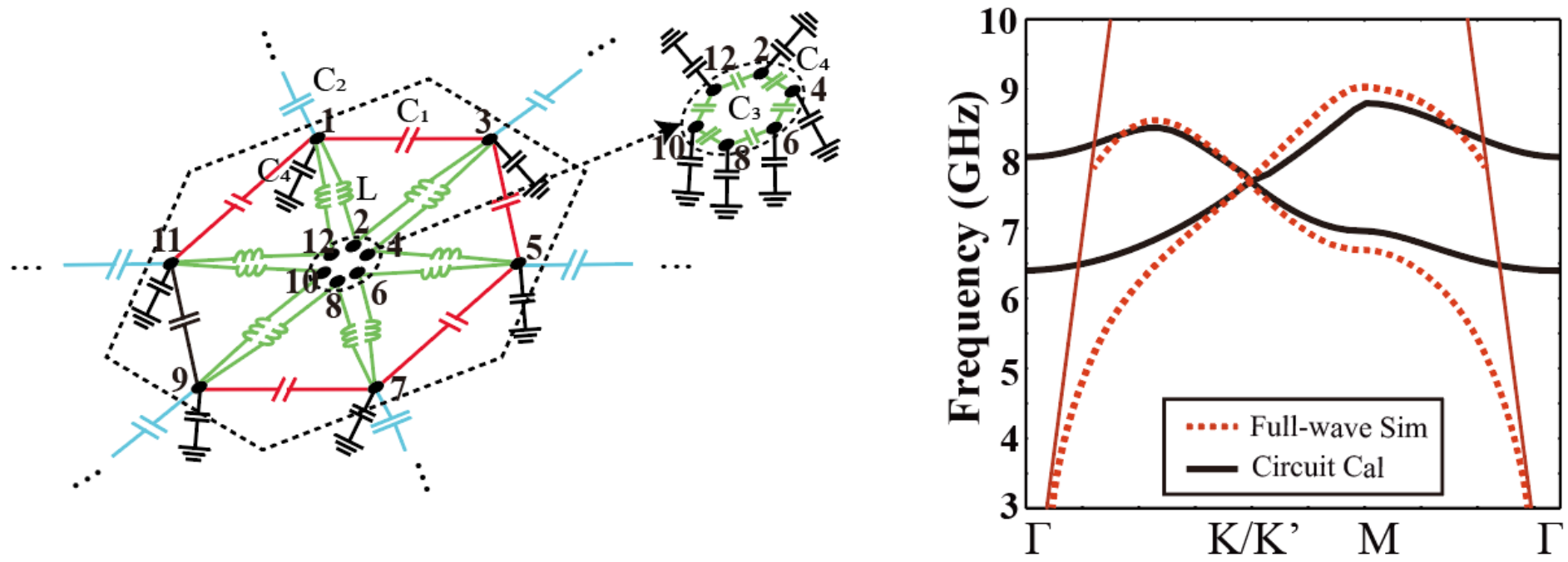

Figure S3. Equivalent circuit model and the calculated bands of the hollow-snowflake-wire structure.

S2. The electric field distributions along z direction of the three metasurfaces

The electric field amplitudes of eigenstates at the Dirac points for the three structures shown in figures 1(c)-(e) are extracted, as shown in figure S4. The normalized electric field amplitudes decay exponentially along Z direction, exhibiting the typical characteristics of sspp modes. Importantly, the hollow-snowflake-wire structure shows the most rapid field decay, in agreement with the band dispersions and the penetration depth results shown in figures 1(c)-(f), demonstrating the strongest vertical confinement.

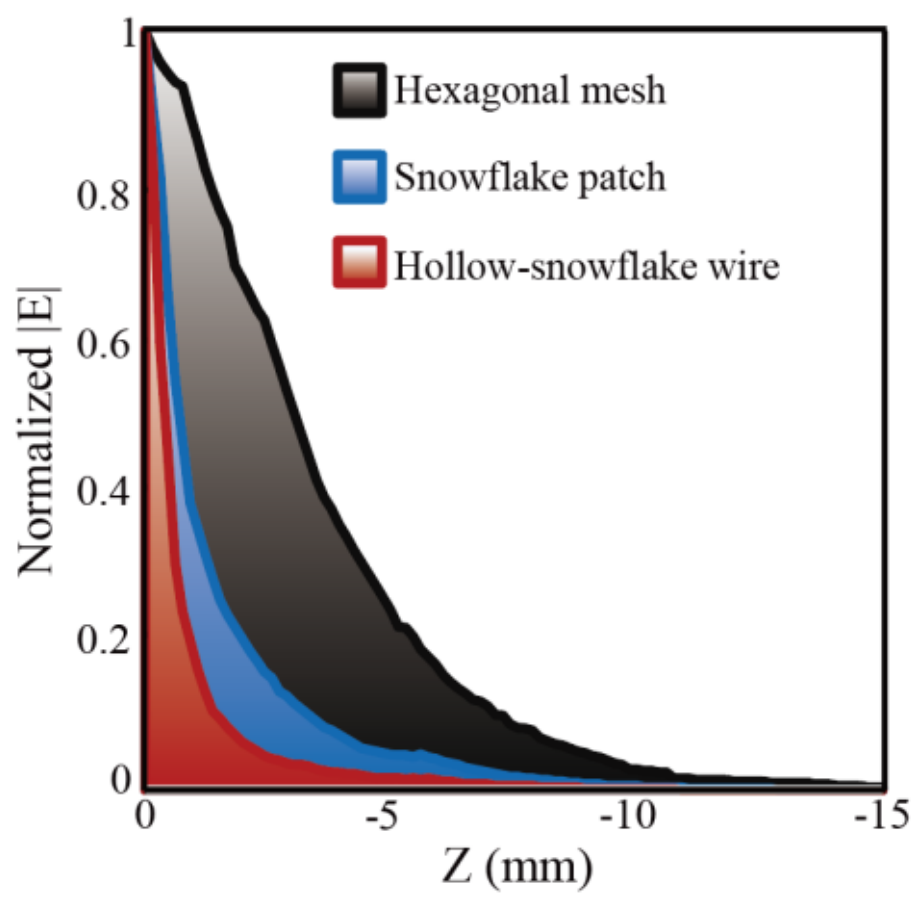

Figure S4. Normalized electric field amplitudes along the Z direction for the eigenstates supported by the three structures.

S3. Relationship between optical transparency and electromagnetic performance

To quantitatively investigate the trade-off between optical transparency and electromagnetic performance, we performed a comparative study for three representative cases with $w_1 = 0.1$, 0.2, and 0.3 mm, while keeping all other geometric parameters unchanged. The corresponding optical transparency and electromagnetic performance are summarized in Table S1.

Table S1. Optical transparency and electromagnetic performance of the transparent DSPC with different metallic wire widths.

w_1 (mm)	Transparency (%)	Operating bandwidth (GHz)	Normalized penetration depth	Averaged metal loss per wavelength (%)
0.3	>75	1.6 (7.4~9.0)	0.17	3.18
0.2	>85	1.0 (7.7~8.7)	0.14	3.87
0.1	>92	0.6 (7.5~8.1)	0.10	4.55

As the metallic wire width decreases from 0.3 mm to 0.1 mm, the metallic area within each unit cell is reduced, resulting in an increase in the optical transparency from over 75% to over 92%. Meanwhile, the thinner metallic wire introduces a larger equivalent inductance, which lowers the eigenfrequencies of the unit cell and shifts the valley bandgap toward lower frequencies. This downward shift is accompanied by a reduction in the operating bandwidth. To quantify the influence of the metallic wire width on field confinement, the normalized penetration depth (defined in the same manner as in Figure 1(f) of the manuscript) was also calculated. As the metallic wire becomes thinner, the electromagnetic field exhibits stronger vertical confinement, leading to a smaller normalized penetration depth. In addition, the propagation loss was evaluated for the three cases. Since the loss tangent of the silica-glass substrate is only 5.7×10^{-5}, the dielectric loss is negligible. Therefore, the propagation loss is dominated by the metallic ohmic loss. A thinner metallic wire results in a higher electrical resistance, while the enhanced mode confinement further increases the surface current density. Both effects contribute to increased ohmic dissipation. To provide a quantitative comparison, the averaged metallic loss per wavelength was calculated, and the results are in good agreement with the above physical interpretation.

Overall, increasing the optical transparency by reducing the metallic wire width enhances the vertical field confinement, but at the expense of a narrower operating bandwidth and higher metallic propagation loss.

S4. Explanation for the eigenfrequency shifting as α_i varying

In figure 2(d), the non-degenerated LCP and RCP eigenstates show up-shifting frequencies as α_i decrease or increase away from 60°. This behavior arises because when $\alpha_i = 60^\circ$, the metal wires in the unit cell remain their original length, leading to the largest equivalent inductance. As α_i decreases, the two branches within each pair increasingly overlap, resulting in a reduction of metal wire length, and thus a reduction of the equivalent inductance between nodes. This effect is most pronounced at $\alpha_i = 0^\circ$, where the two branches fully overlap and the equivalent metal length is minimized as shown in figure S5. In the equivalent circuit model, the decrease in inductance leads to an increase in the eigenfrequency, thereby giving rise to the trend observed in figure 2(d).

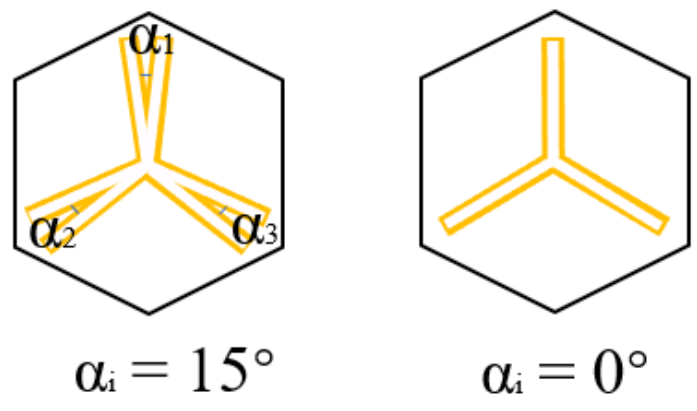

Figure S5. The profiles of unit cell with $\alpha_i = 15^\circ$ or $\alpha_i = 0^\circ$.

S5. Spatial Fourier spectra of the topological power splitter and diplexer

To directly verify the directional propagation of valley-polarized interface and surface states (VISs and VSSs) without inter-valley scattering, we extract the simulated complex Ez components of the power splitter in Figure 3(d) and the diplexer in Figure 5(b), and perform spatial Fourier transforms on the interior regions (indicated by the green dashed rectangles in Figure S6). The corresponding Fourier spectra are plotted below, with the green solid hexagons denoting the first Brillouin zones. In all three cases, the spectra exhibit dominant momentum components at the K' points, confirming that both VISs and VSSs propagate robustly across interconnects and transformations without inter-valley scattering. It should be noted that the field components are extracted at a plane 2 mm above the structure surface, which introduces the propagation mode in air and consequently results in additional components appearing in the Fourier spectra. The VSSs exhibit a little more radiation leakage in transverse directions than VIS, thus leading to a slightly broadened k-space distribution.

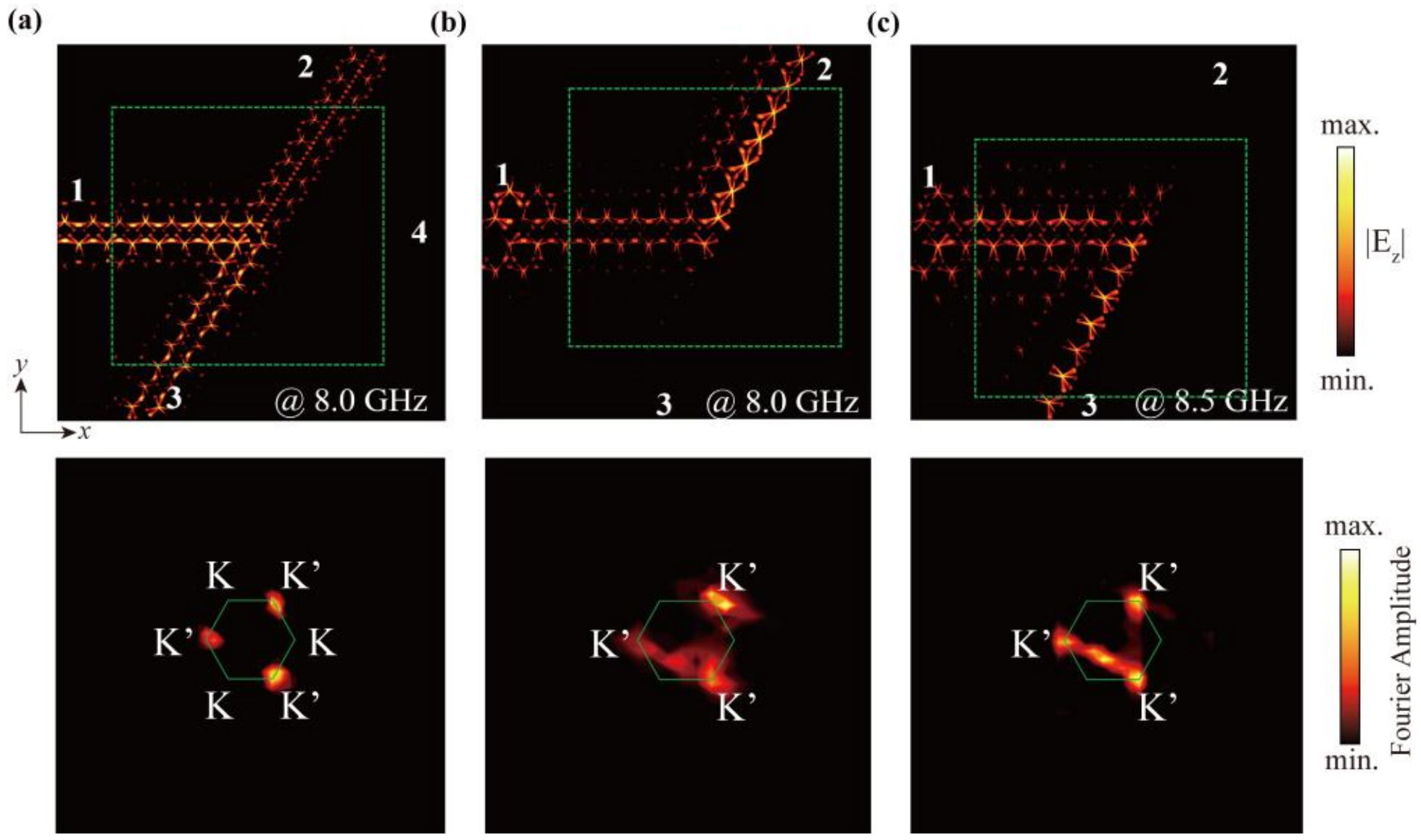

Figure S6. Spatial Fourier spectra of VIS and VSSs. The green dashed rectangles mark the interior regions used for Fourier transforms, and the green solid hexagons indicate the first Brillouin zones. (a) Simulated field distribution of the power splitter in Figure 3(d) and its Fourier spectrum. (b, c) Simulated field distributions of the topological diplexer in Fig. 5(b) and their corresponding Fourier spectra.

S6. Equivalent circuit calculation of the tailorable valley-polarized surface states

The valley-polarized surface state bands can also be calculated based on the equivalent circuit model. Five unit cells arranged in the transverse direction are selected to construct a supercell, and then a

circuit model with 60 nodes is obtained. Based on the generalized eigenvalue formulation, we construct the capacitance and inductance matrix with 60×60 sizes and calculate the surface state bands as shown in figure S7.

In the left panel, the supercell band structure is obtained when the boundary unit is chosen as Unit III with varying α_i. In this case, the capacitance between sites 1 and 3 is set to $C_1' = C_1/\text{ratio}$, while the capacitances between sites 1–11 and sites 3–5 are modified to $C_2' = C_2 \times \text{ratio}$. As the ratio increases, the surface band gradually shifts upward in frequency, which is consistent with the trend observed in Figure 4(b), where the surface band moves upward with increasing α_1.

Similarly, the right panel shows the supercell band structure when the boundary unit is Unit IV with varying β_j. Here, the capacitance between sites 1 and 3 is increased to $C_2' = C_2 \times \text{ratio}$, while the capacitances between sites 5–7 and sites 9–11 are reduced to $C_2' = C_2/\text{ratio}$. The results again demonstrate that the surface band shifts upward as the ratio increases, in excellent agreement with the behavior reported in Figure 4(b). These observations confirm the validity and accuracy of the equivalent circuit model in describing the surface-band modulation of the hollow-snowflake-wire structure.

Owing to its high computational efficiency and flexibility in parameter tuning, the equivalent circuit model serves as an effective design tool for guiding the development of topological units and the associated surface bands. By leveraging the circuit-based representation of the structure, the reliance on computationally expensive full-wave simulations can be substantially reduced, thereby significantly lowering the overall design cost.

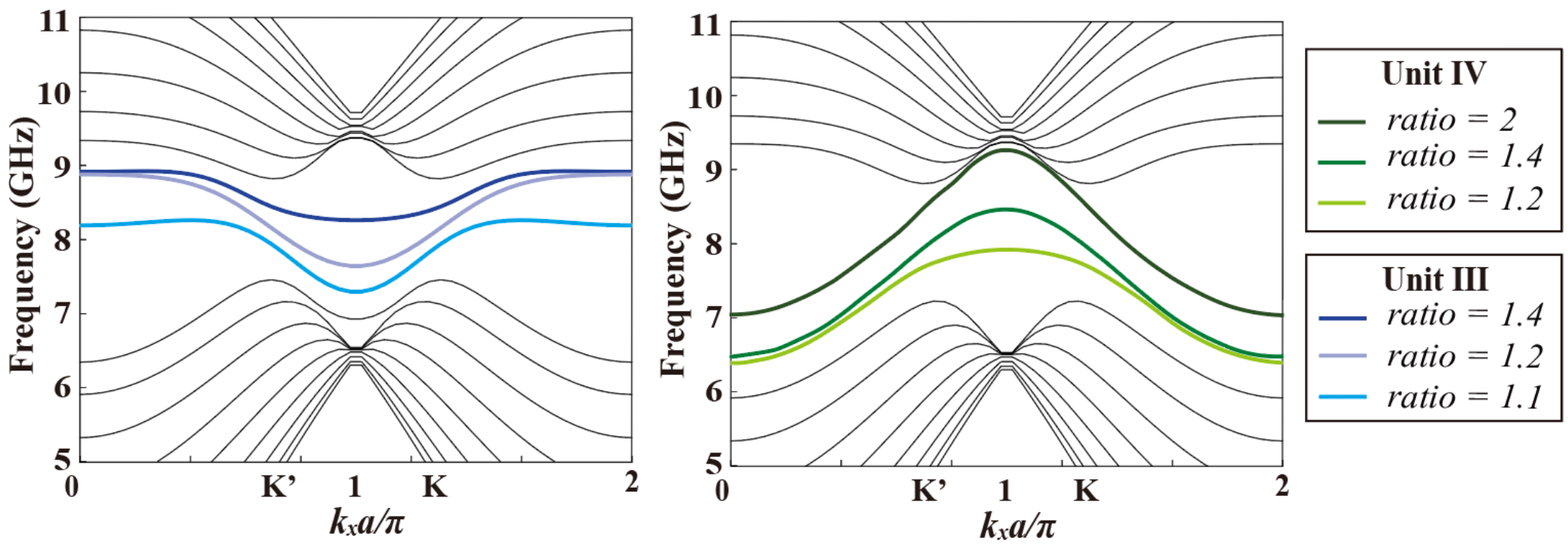

Figure S7. Tailorable surface bands calculated by the equivalent circuit models. Three blue (green) lines represent the surface bands located at the boundaries formed by Unit III (Unit IV) with varying $\alpha_1(\beta_j)$. The parameter *ratio* represents the capacitor changes according to the structure modification with varying $\alpha_1$and β_j in figure 4.

S7. The profiles of perturbed boundary unit cells

The band diagrams of VSSs with tailored boundary unit cells are shown in figure 4(b). To provide a clearer view of the corresponding structures under different parameters, the four residual perturbed unit cells not presented in the main text are plotted in figure S8. The unit cells modified from unit I with variations in α_1 only are displayed in figure S8(a), whereas the unit cells modified from unit II with varying β_j $(j = 2, 3)$ are shown in figure S8(b).

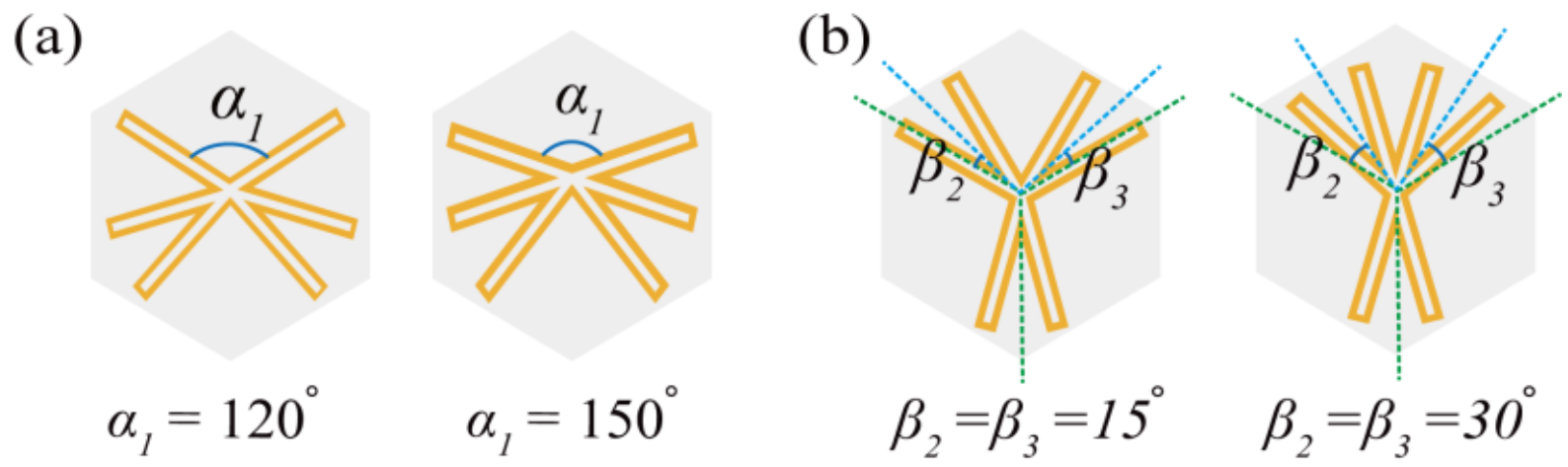

Figure S8. Structure profiles of the adjusted surface unit cells. (a) Deformed boundary unit cells based on Unit I with α_1 varying only. (b) Deformed boundary unit cells based on Unit II, where β_j $(j = 2, 3)$ vary with other parameters unchanged.

S8. The quantitative robustness performance against local defects of VIS and VSS

The straight VIS and VSS waveguides constructed from TDSPCs A and D are illustrated in Figures. S9(a) and S9(d), respectively, where the intentionally introduced local defects are highlighted by the red dashed circles. The corresponding electric-field distributions in Figures. S9(b) and S9(e) demonstrate efficient wave propagation across the defective regions. Furthermore, the normalized transmission ratios in Figures. S9(c) and S9(f) show that the transmission characteristics of the defective waveguides closely match those of the corresponding defect-free structures. These results confirm that both VISs and VSSs exhibit excellent propagation robustness against local defects.

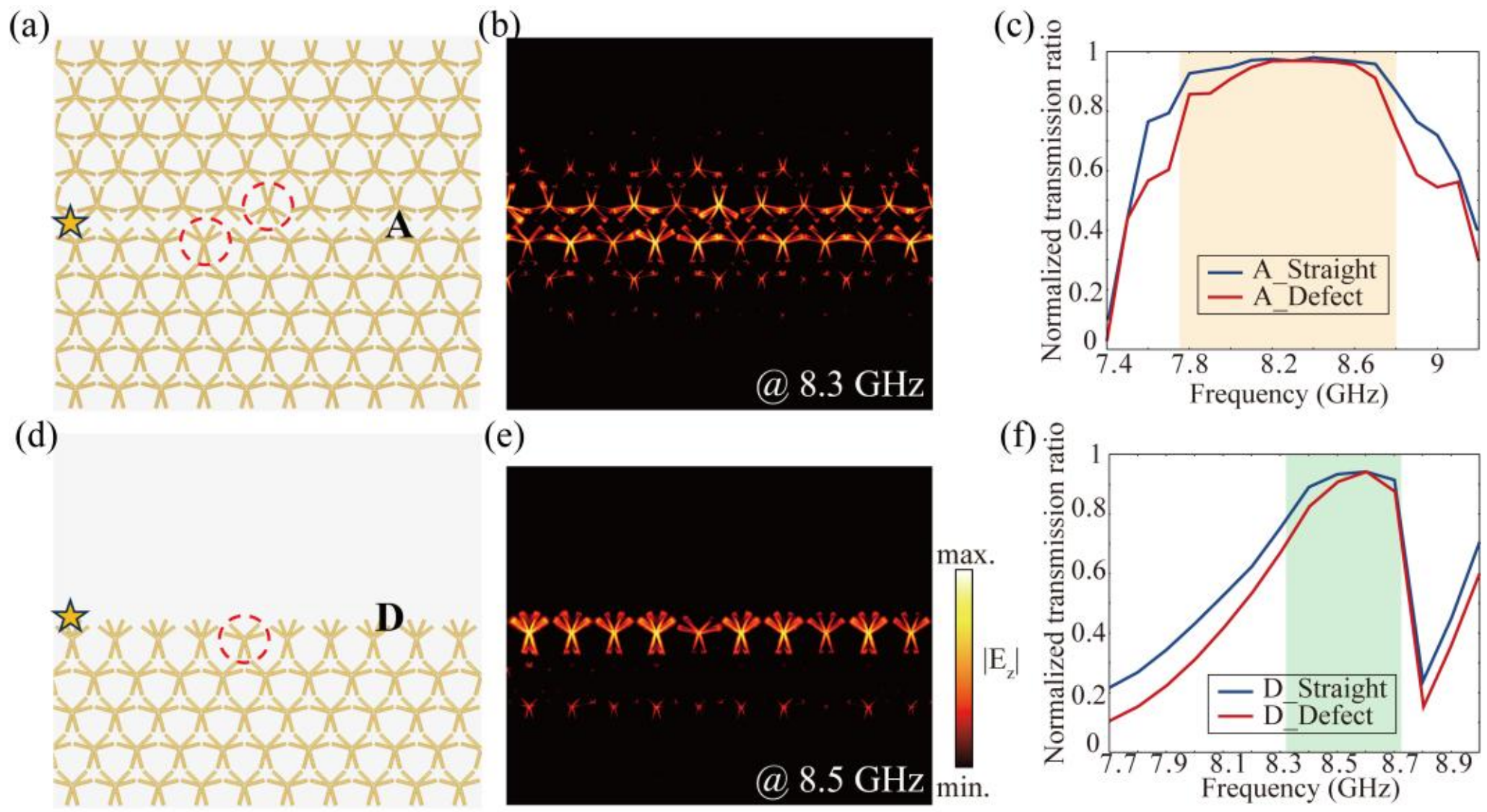

Figure S9. Robust propagation of valley interface states (VISs) and valley surface states (VSSs) against local defects. (a,d) Straight VIS and VSS waveguides with intentionally introduced local defects (red dashed circles). (b,e) Simulated electric-field distributions for both waveguides with defects. (c,f) Normalized transmission ratios for the perfect and defective waveguides.

S9. The Ez field probe for near-field measurement

The detector used in our near-field measurement is an ultra-wideband electric field probe with high sensitivity [2]. It features a bandwidth from 10 MHz to 60 GHz and a spatial resolution of 0.7 mm, enabling accurate field measurement for our diplexer sample.

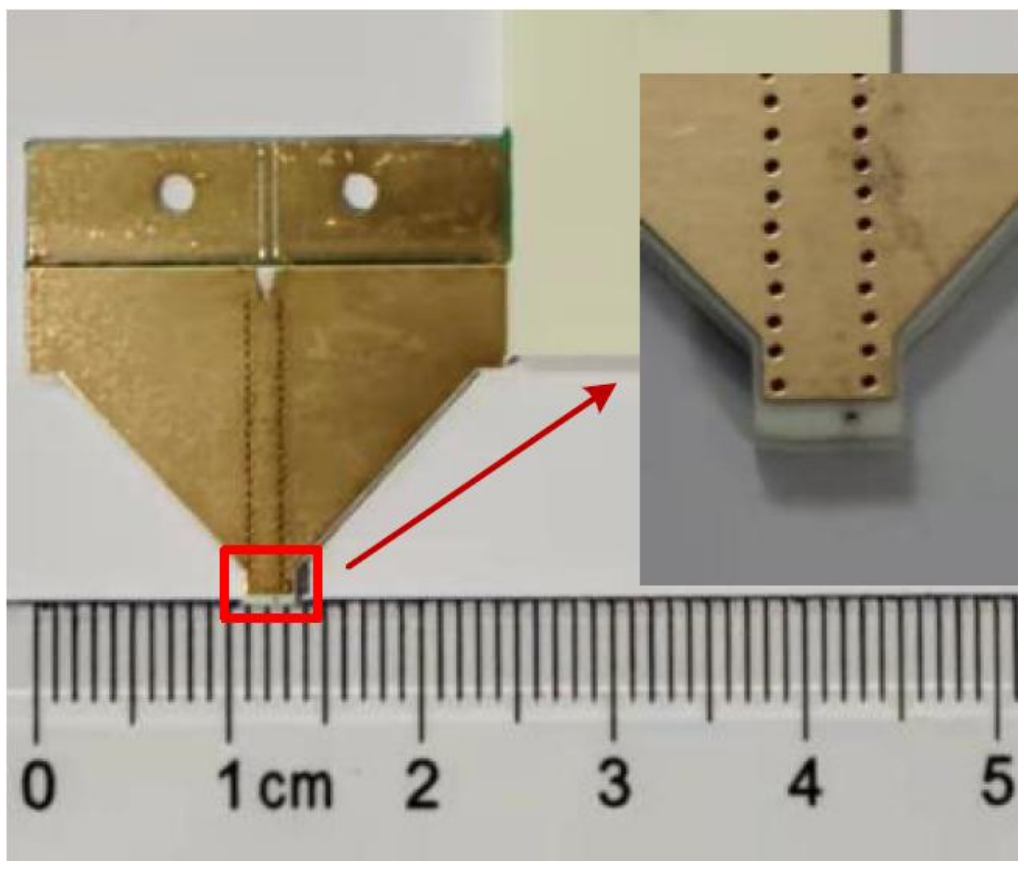

Fig. S10. The photography of the electric field probe for near-field measurement [2].

S10. Fabrication tolerances of the transparent DSPC

The proposed transparent DSPC is fabricated using a standard photolithography and wet-etching process on a copper-coated silica substrate. In practice, the dominant fabrication deviation is a uniform line-width bias caused by variations in exposure, photoresist development, and metal etching. Such a deviation simultaneously changes the inter-wire gaps (illustrated by w_1 and w_2 in Figure 2 of the manuscript) and therefore represents the most common fabrication tolerance in photolithographic processing.

To evaluate the fabrication tolerance of the proposed design, we performed a full-wave analysis by introducing a ±10% uniform variation in the metallic wire width. Specifically, w_1 was varied from the nominal value of 0.20 mm to 0.18 mm and 0.22 mm, while the corresponding inter-wire gap w_2 changed to 0.24 mm and 0.16 mm, respectively, with all other geometric parameters unchanged. The resulting valley bandgaps and diplexer performance are presented in Figures S11 and S12.

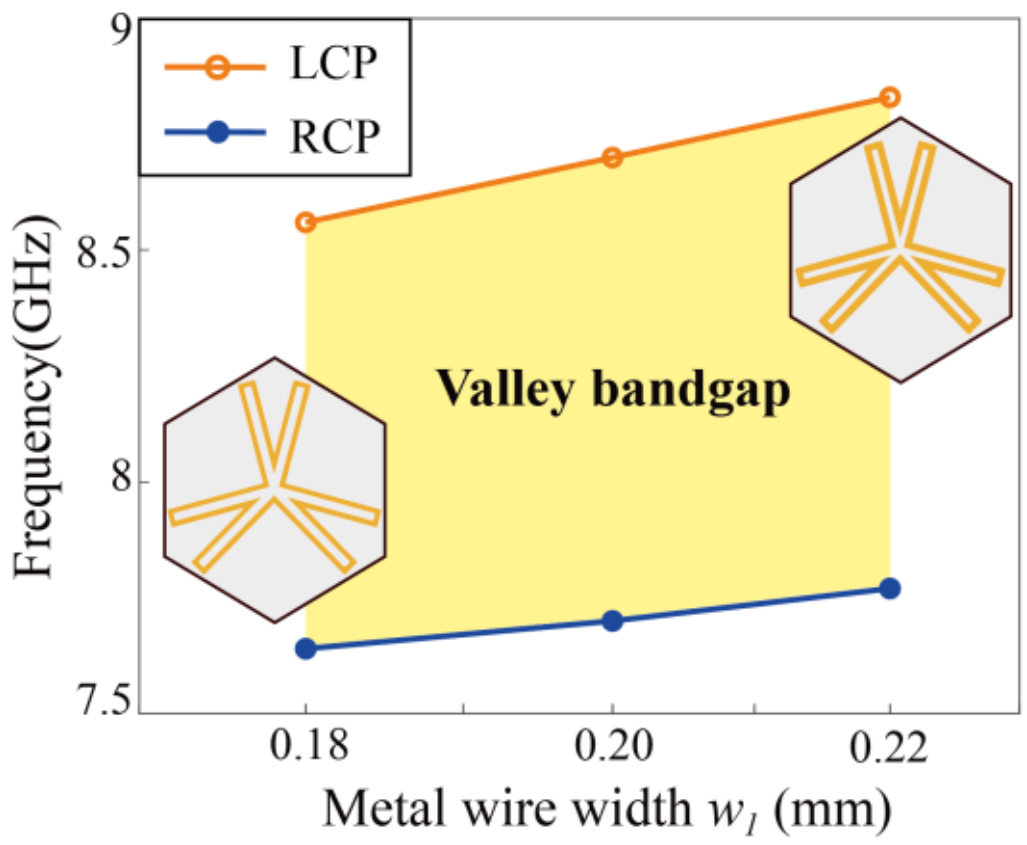

Figure S11. The valley bandgaps for disturbed unit cells.

As shown in Figure S11, decreasing the metallic wire width increases the equivalent inductance, shifting the valley bandgap downward from 7.70–8.69 GHz to 7.64–8.56 GHz, whereas increasing the wire width produces the opposite effect, shifting the bandgap upward to 7.77–8.83 GHz. Correspondingly, the diplexer operating bands exhibit the same frequency shifts. As shown in Figures S12(b) and S12(d), more than 90% of the input power is still routed to Port 2 and Port 3 within the

corresponding lower- and upper-band operating ranges, respectively, which are in good agreement with the calculated valley bandgaps. The electric-field distributions in Figures S12(a) and S12(c) further confirm that the diplexer maintains the desired frequency-selective routing behavior under both tolerance cases.

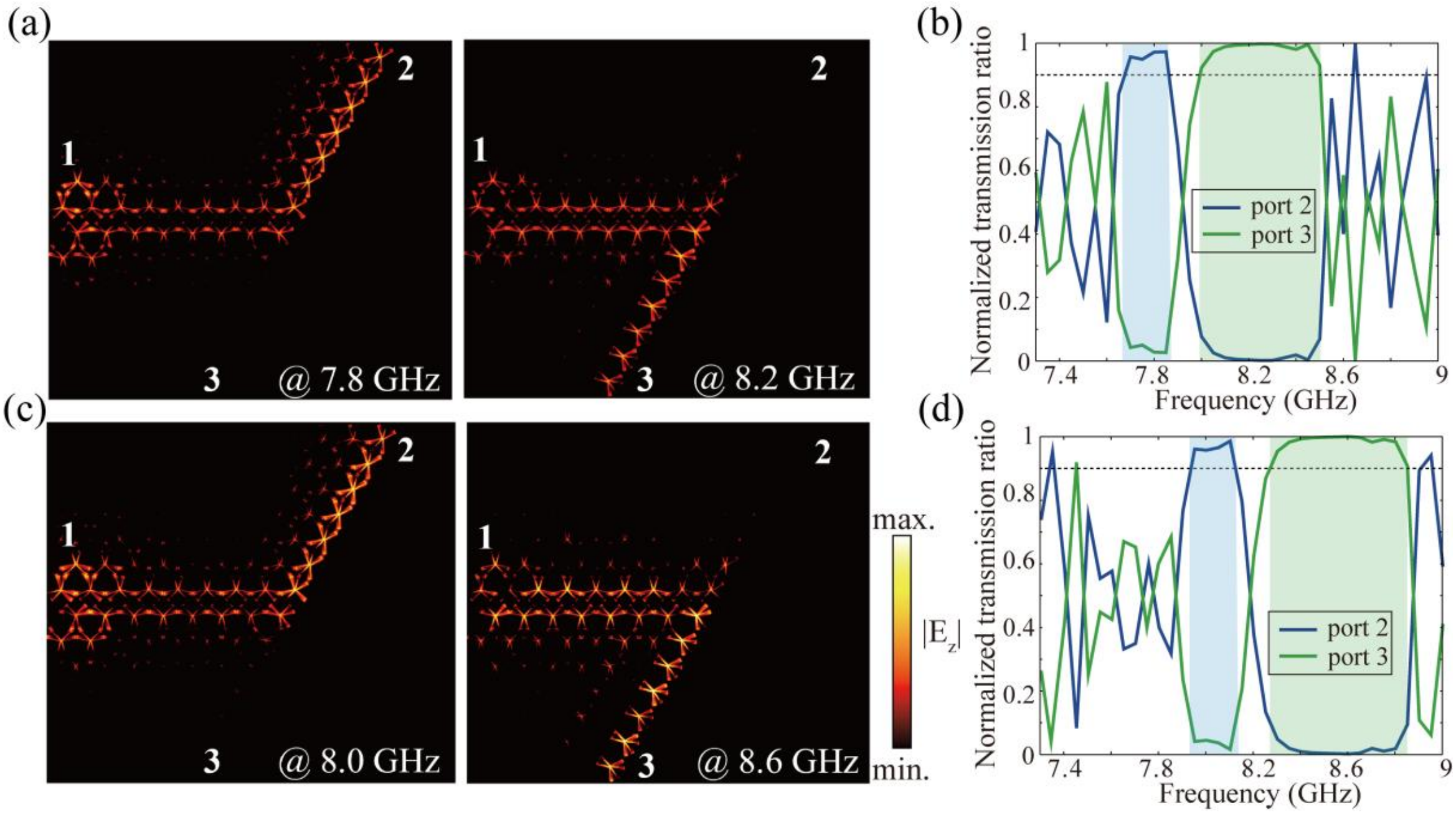

Figure S12. Operation performance of the topological diplexer with biased structural parameters. (a) The simulated electric field maps and (b) normalized transmission ratio of the diplexer with $w_1 = 0.18mm$ and $w_2 = 0.24mm$. (c) The simulated electric field maps and (d) normalized transmission ratio of the diplexer with $w_1 = 0.22mm$ and $w_2 = 0.16mm$.

Overall, the valley bandgap remains open under both ±10% fabrication deviations, indicating that the designed C_{3v}-symmetry breaking and the underlying topological phase are preserved. Consequently, typical variations in the metallic wire width and inter-wire gap only cause slight shifts in the operating frequency, while the valley interface states and diplexer functionality remain essentially unchanged. These results demonstrate the high fabrication tolerance and practical manufacturability of the proposed transparent topological metasurface.

S11. A frequency-dependent topological router with unidirectional-excitation property

By introducing a tapered air gap into the valley bulk region composed of Unit I, a structure consisting of two gradually decoupled surface waveguides and a common termination is constructed as shown in Figure S13(a). The device comprises three sections: part I is a homogeneous valley bulk, functioning as a termination due to its bulk bandgap; part II is an air region with a linearly increasing width (from 0 to 1.2a) inserted into the valley bulk; and part III contains two surface waveguides arranged head-to-head, decoupled by an air gap with a fixed width of 1.2a. We extract the two surface waveguides in part III, referred to as TDSPCs E and F, and calculate their dispersion diagrams as illustrated in Figure S13(b). Each TDSPC consists of a single valley bulk region with air filling the opposite side, and supports one valley-polarized surface state (VSS) band within the bandgap.

Exploiting the separated frequency ranges of the two VSS bands, the structure in Figure S13(a) achieves frequency-selective routing with strong inter-channel isolation. As shown in Figure S13(c), the excitation source, marked by the yellow pentagram, is placed in the air region of Part II. The field distributions reveal that the excited waves terminate at the interface between Part I and Part II, and propagate rightward along either the upper or lower surface boundary, depending on their frequency band. No coupling or interference between the two VSSs is observed, indicating strong isolation from neighboring modes. Moreover, this terminating effect enabled by the gradual transition cannot be achieved in conventional VIS-based structures, since the topological phase transition of the units requires a closed bandgap where interface states emerge to transport energy.

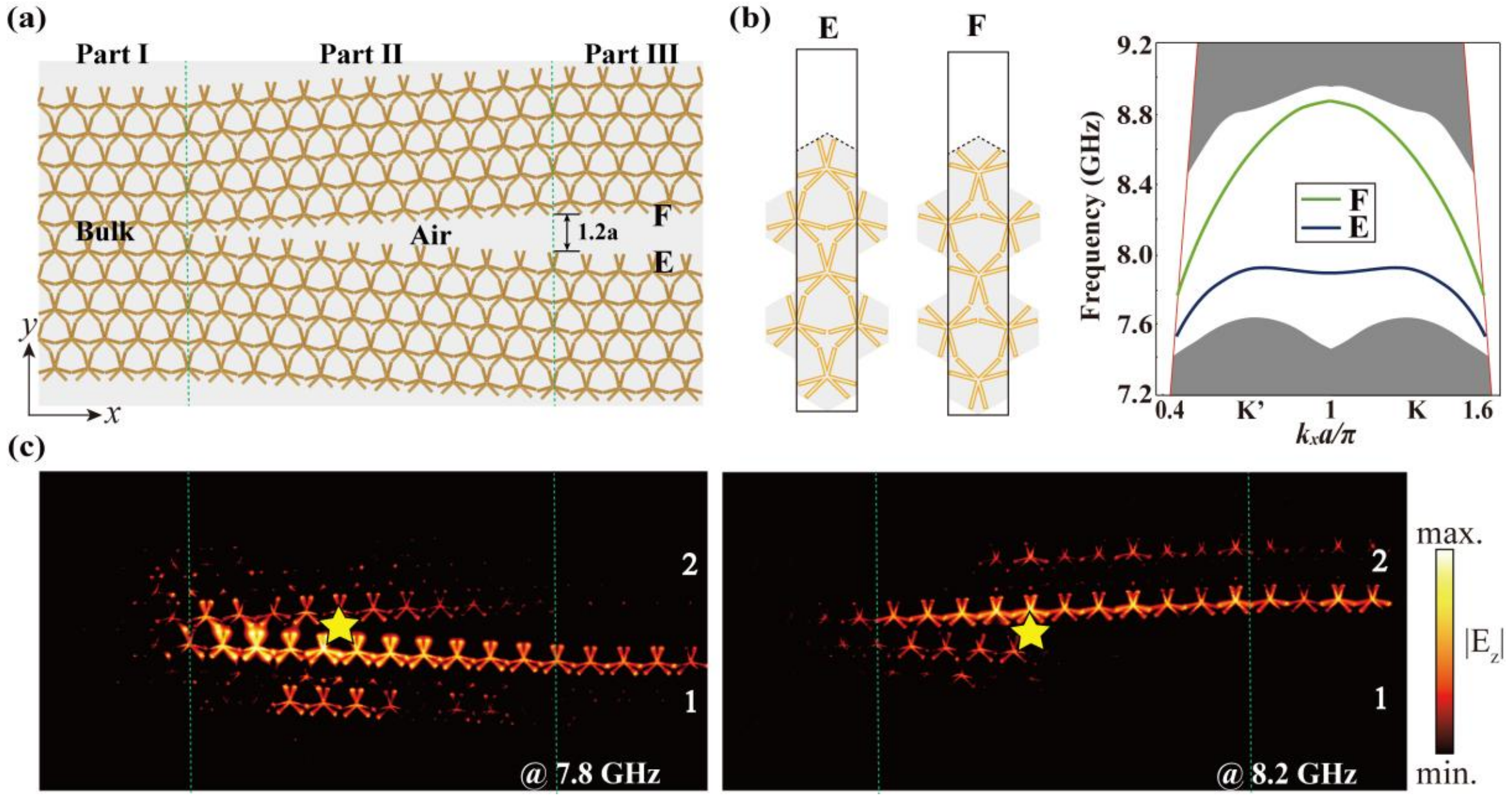

Figure S13. Unidirectionally-excited topological frequency router based on valley-polarized surface states (VSSs). (a) Introduction of a tapered air gap (width from 0 to 1.2a) within a single valley bulk domain. The air gap decouples the upper and lower boundaries, labeled F and E, respectively. (b) Profiles of the corresponding TDSPCs E and F with their band diagrams supporting the VSSs. (c) Electric field distributions of the excited VSSs corresponding to distinct frequencies. The air gap enables two independent VSSs in spectrally separated bands. The field maps show the rightward-propagating VSSs localized at either the upper or lower boundary, operating in distinct frequency ranges without mutual interference.

S12. Another topological diplexer configuration

Benefit from the tightly-confined valley-polarized surface states, the inter-channel isolation is strongly prohibited and the device footprint is much more compact, contributing to flexible designs for topological devices. As shown in figure S14, another topological meta-diplexer is constructed by removing two rows of unit cells adjacent to the interface domain wall of TDSPC A, thereby replacing two surface boundaries with TDSPC C and D above and below the central air region, respectively. Since TDSPCs C and D support VSSs covering different frequency ranges, the energy excited as VIS A selectively couples to either VSS C or VSS D depending on the operating frequency, same with the diplexer in figure 5(a). The state transition occurs smoothly at the interconnection point with minimal loss. Notably, despite the close spacing between the two surface waveguides, no mutual coupling or

interference arises, enabling a topological diplexer featuring strong isolation but an ultra-compact configuration that can't be accomplished by conventional VIS-based designs.

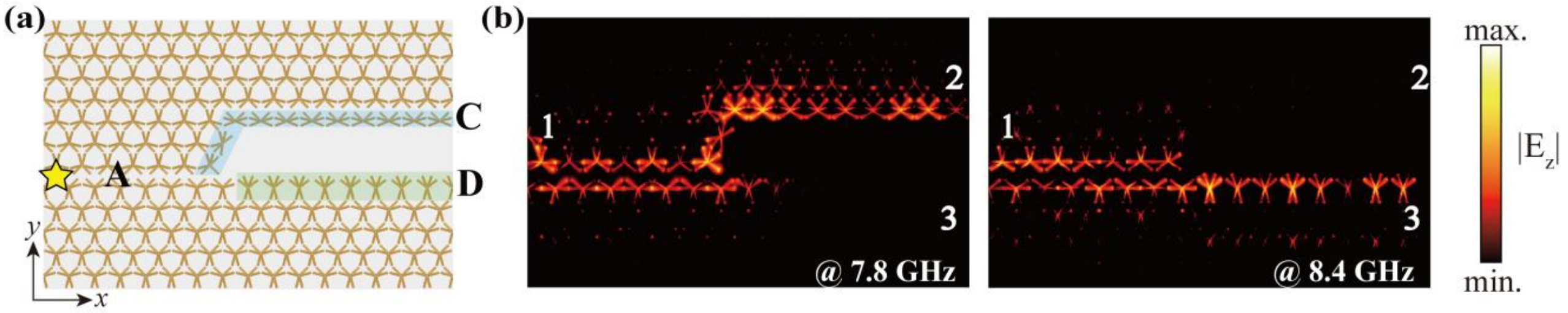

Figure S14. Schematics and the simulated field distributions of another topological diplexer. (a) The profile of the topological diplexer. (b) The simulated field distributions show the efficient frequency-dependent routing without inter-channel interaction.

S13. IQ constellation diagrams for varying symbol rates under 64-QAM modulation

In the communication experiment, we transmit 64-QAM signals with carrier frequencies of 8.1 GHz or 8.5 GHz using VSG. The symbol rates vary from 1 Msyms/s to 20 Msyms/s, and the corresponding in-phase and quadrature (IQ) constellation diagrams of received signals at Port 2 and Port 3 are recorded in figure S15. As the symbol rate increases, the constellation performance degrades as expected. However, even at 20 Msym/s, the constellation points remain well separated, and the received data can still be clearly demodulated and reliably analyzed, well consistent with the EVM results shown in figure 6(d).

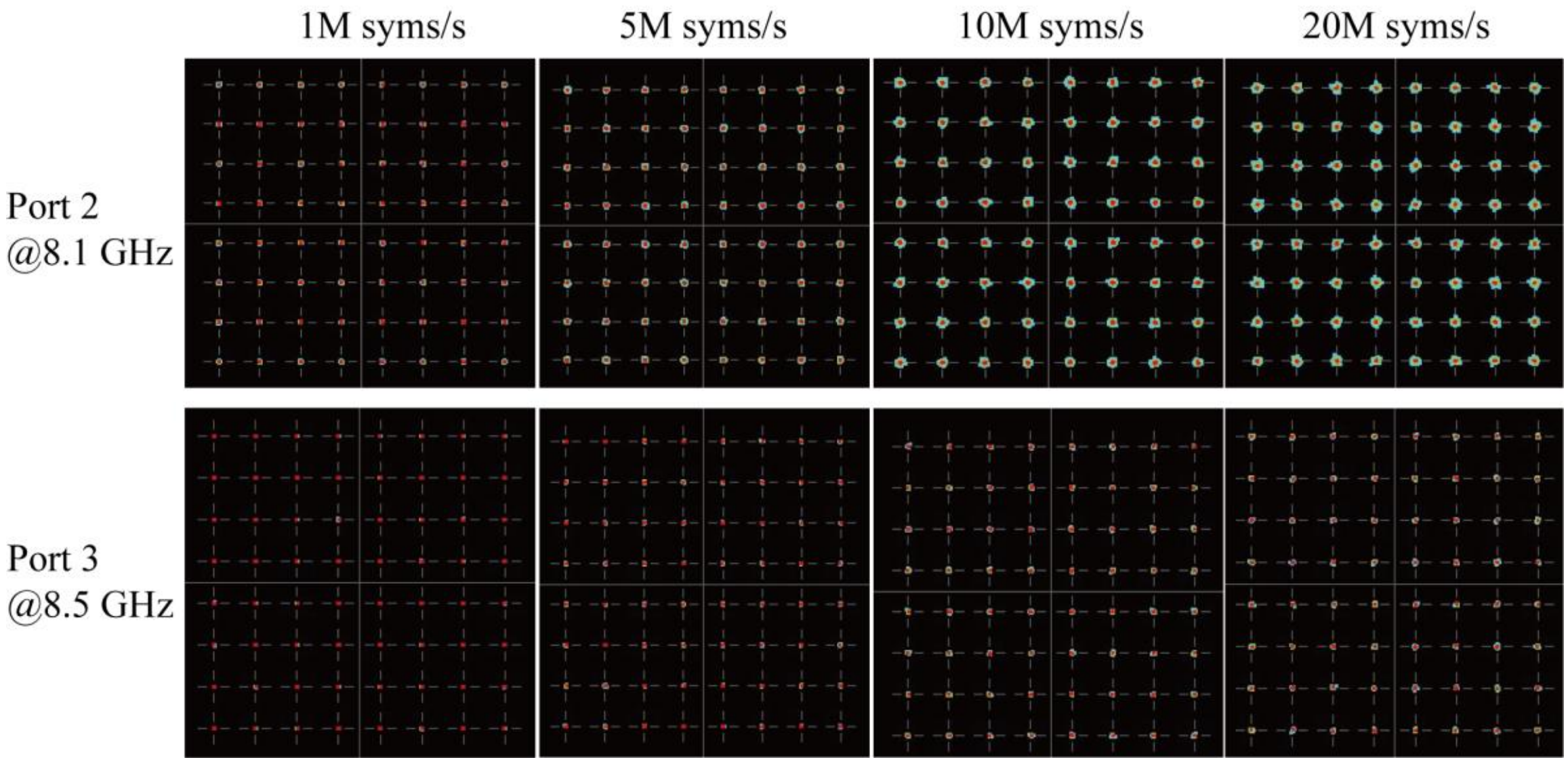

Figure S15. The in-phase and quadrature (IQ) constellation diagrams of the two output ports under 64-QAM modulation with symbol rates from 1 Msym/s to 20 Msym/s.

S14. Data stream communication performance test

To experimentally verify the data transmission capability of our meta-diplexer, a short bitstream consisting of 112 binary digits was used as the input signal. The signal was QPSK-modulated by a vector signal generator (VSG) at a symbol rate of 1 Msym/s and injected into the diplexer. The output signals were received at Port 2 (8.1 GHz) and Port 3 (8.5 GHz), and subsequently demodulated using

a vector signal analyzer (VSA). Figures S16(a) and S16(b) present the IQ constellation and eye diagrams, respectively, both demonstrating robust and accurate transmission performance. The demodulated bitstream is shown in Fig. S16(c), where each interval between two adjacent red markers corresponds to one data stream period. The results indicate that the transmitted data are precisely captured, thereby confirming the practical data transmission capability of the proposed meta-diplexer.

Furthermore, a total of 480,000 bits of randomly generated data were transmitted, yielding a measured bit error rate (BER) of zero, which further validates the accuracy of the EVM results.

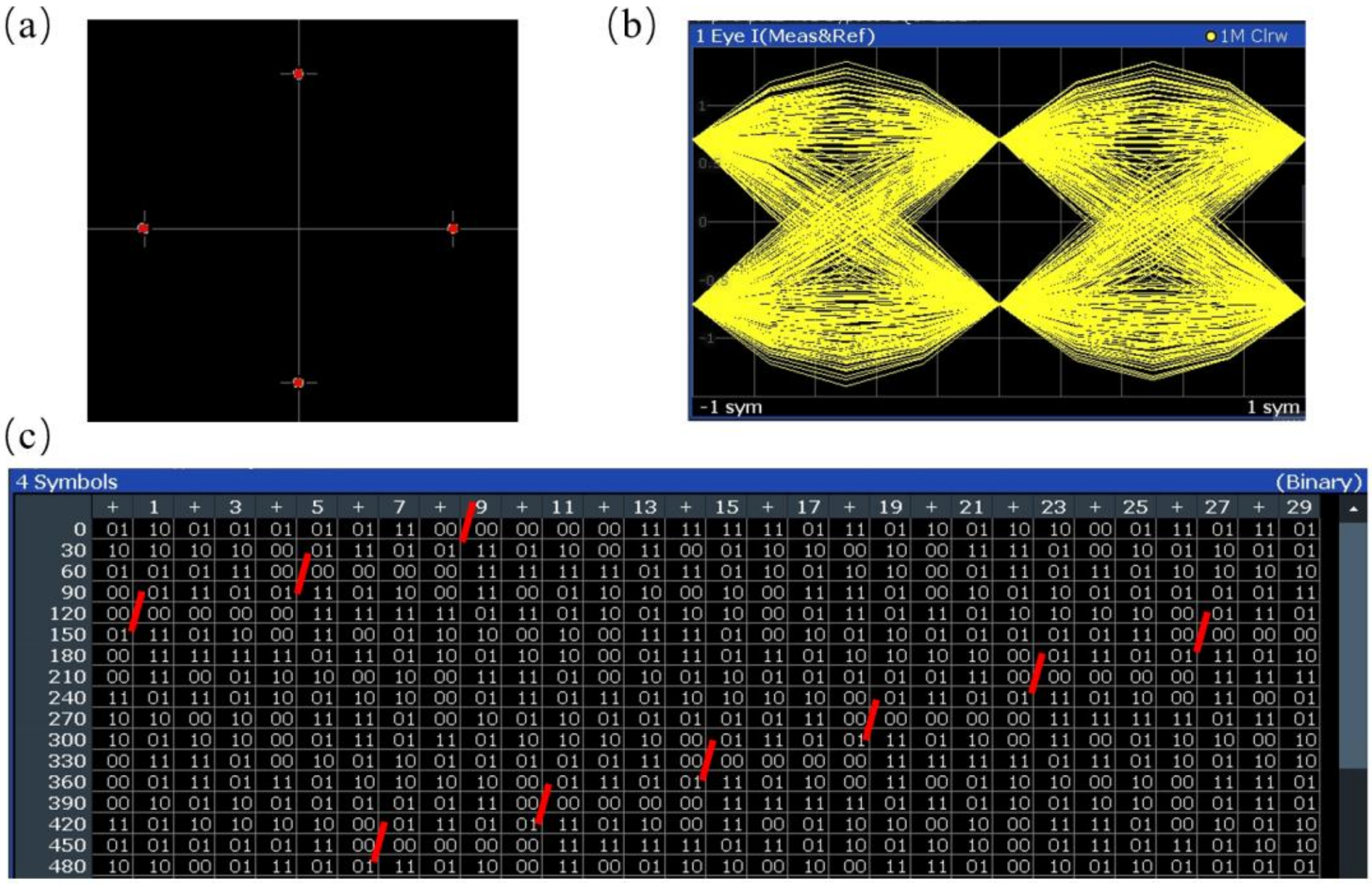

	+	1	+	3	+	5	+	7	+	9	+	11	+	13	+	15	+	17	+	19	+	21	+	23	+	25	+	27	+	29
0	01	10	01	01	01	01	01	11	00	00	00	00	00	11	11	11	11	01	11	01	10	01	10	10	00	01	11	01	11	01
30	10	10	10	10	00	01	11	01	01	11	01	10	00	11	00	01	10	10	00	10	00	11	11	01	00	10	01	10	01	01
60	01	01	01	11	00	00	00	00	00	11	11	11	11	01	11	01	10	01	10	10	00	01	11	01	11	01	10	10	10	10
90	00	01	11	01	01	11	01	10	00	11	00	01	10	10	00	10	00	11	11	01	00	10	01	10	01	01	01	01	01	11
120	00	00	00	00	00	11	11	11	11	01	11	01	10	01	10	10	00	01	11	01	11	01	10	10	10	10	00	01	11	01
150	01	11	01	10	00	11	00	01	10	10	00	10	00	11	11	01	00	10	01	10	01	01	01	01	01	11	00	00	00	00
180	00	11	11	11	11	01	11	01	10	01	10	10	00	01	11	01	11	01	10	10	10	10	00	01	11	01	01	11	01	10
210	00	11	00	01	10	10	00	10	00	11	11	01	00	10	01	10	01	01	01	01	01	11	00	00	00	00	00	11	11	11
240	11	01	11	01	10	01	10	10	00	01	11	01	11	01	10	10	10	10	00	01	11	01	01	11	01	10	00	11	00	01
270	10	10	00	10	00	11	11	01	00	10	01	10	01	01	01	01	01	11	00	00	00	00	00	11	11	11	11	01	11	01
300	10	01	10	10	00	01	11	01	11	01	10	10	10	10	00	01	11	01	01	11	01	10	00	11	00	01	10	10	00	10
330	00	11	11	01	00	10	01	10	01	01	01	01	01	11	00	00	00	00	00	11	11	11	11	01	11	01	10	01	10	10
360	00	01	11	01	11	01	10	10	10	10	00	01	11	01	01	11	01	10	00	11	00	01	10	10	00	10	00	11	11	01
390	00	10	01	10	01	01	01	01	01	11	00	00	00	00	00	11	11	11	11	01	11	01	10	01	10	10	00	01	11	01
420	11	01	10	10	10	10	00	01	11	01	01	11	01	10	00	11	00	01	10	10	00	10	00	11	11	01	00	10	01	10
450	01	01	01	01	01	11	00	00	00	00	00	11	11	11	11	01	11	01	10	01	10	10	00	01	11	01	11	01	10	10
480	10	10	00	01	11	01	01	11	01	10	00	11	00	01	10	10	00	10	00	11	11	01	00	10	01	10	01	01	01	01

Figure S16. Specific data stream transmission test under QPSK modulation. (a) IQ constellation diagram for the received signal. (b) Eye diagram of the output port. (c) Portion of the demodulated data stream captured from the VSA interface, where red short lines indicate the boundaries between successive data stream periods.

References

[1] S. Liu, W. Gao, et al. Topologically protected edge state in two-dimensional Su-Schrieffer-Heeger circuit. Research, 2019.
[2] Y. X. Liu, X. C. Li, et al. An ultra-wideband electric field probe with high sensitivity for near-field measurement. IEEE Transactions on Instrumentation and Measurement, 2023, 72: 1-10.

S15. Quantitative comparison of the topological diplexers/multiplexers

To better highlight the advantages of the proposed device, we have added a quantitative comparison with six representative topological multiplexers published within the past five years. These works cover several representative topological platforms, including all-silicon valley photonic crystals (VPCs), metallic gap-waveguide-based VPCs, and microstrip-based VPCs, operating from the microwave to the terahertz regime.

Since these devices are implemented on different physical platforms and operate at different

frequencies, direct comparison of their absolute footprint and substrate thickness is not meaningful. Therefore, for a fair comparison, the footprint and substrate thickness are normalized by the free-space wavelength (λ_0) at the operating frequency, while the operating bandwidth is characterized by the fractional bandwidth (FBW). Other key performance metrics, including optical transparency, routing flexibility, and communication verification, are also summarized. The detailed comparison is presented in the following Table S2.

Table S2. Performance comparison with reported topological multiplexers.

Refs	Topological platform	Normalized footprint (λ_0^2)	Normalized thickness (λ_0)	Optical transparency (%)	FBW (%)	Routing flexibility	Communication verification
[1]	Si VPC	139.20	0.23	opaque	4.12	Fixed	16-QAM, 40Gbit/s
[2]	Si VPC	287.25	0.21	opaque	9.58	Fixed	32-QAM, 150Gbit/s
[3]	Si VPC	249.09	1.43	opaque	1.08	Fixed	QPSK, 1.875Tbit/s
[4]	Metallic VPC	9.88	0.07	opaque	6.67	Limited	None
[5]	Microstrip-type VPC	28.88	0.03	opaque	7.30	Fixed	None
[6]	Microstrip-type VPC	63.72	0.05	opaque	12.20	Fixed	None
This work	Transparent DSPC	**4.93**	**0.03**	**>85**	9.64	**Flexible**	64-QAM, 20 Msym/s

As summarized in Table S2, the proposed topological diplexer simultaneously combines an ultra-compact normalized footprint, an ultrathin substrate, and high optical transparency, while maintaining flexible routing capability and experimentally verified microwave communication performance. To the best of our knowledge, this is the first topological multiplexer that simultaneously integrates these characteristics, demonstrating the unique advantages of the proposed transparent topological platform for practical microwave communication applications.

Refs:

[1] A. Kumar, M. Gupta, P. Pitchappa, et al., Nature Communications 2022, 13, 1 5404.

[2] M. Gupta, A. Kumar, P. Pitchappa, et al., Advanced Materials 2024, 36, 19 2309497.

[3] Z. Guan, K. Wen, C. Xie, et al., APL Photonics 2025, 10, 3.

[4] G. Guo, H. Wang, Q. Wang, et al., Optics Express 2025, 33, 5 12240-12252.

[5] H. Wang, H. Shi, E. I. Wei, et al., IEEE Transactions on Antennas and Propagation 2024, 72, 11 8802-8809.

[6] H. Wang, H. Shi, Z. Lan, et al., Laser & Photonics Reviews 2026, 20, 10 e03173.

S16. Mesh convergence study of the hollow-snowflake-wire geometry

Considering the sharp metallic vertices and narrow metallic strips in the hollow-snowflake geometry,

special attention was paid to the mesh convergence to exclude possible numerical artifacts associated with field singularities. We have therefore performed a systematic mesh convergence study for the C_{3v}-symmetric valley topological unit cell by varying the maximum element size and evaluating the influence of mesh element number on the calculated eigenfrequencies. The detailed results are summarized in the following Table S3 and Figure S17.

Table S3. Mesh convergence study for the valley topological unit I

Maximum element size (mm)	15.5	10.5	6.3	4.0	3.2	2.1	1.7	1.2
Mesh element number	1578	1712	2470	3031	4032	5917	7659	11600
LCP eigenfrequency (GHz)	9.02	9.35	8.88	8.79	8.67	8.69	8.68	8.69
RCP eigenfrequency (GHz)	7.34	7.96	7.78	7.72	7.68	7.70	7.70	7.70

As shown in Figure S17, the calculated eigenfrequencies exhibit slight fluctuations during the initial mesh refinement process. This behavior originates from the discretization error of the finite-element method, where different mesh resolutions lead to different numerical approximations of the electromagnetic eigenmodes. With further mesh refinement, the variation of the calculated eigenfrequency gradually decreases, and the results become converged when the mesh element number exceeds approximately 6000. The obtained convergence behavior confirms the reliability and numerical accuracy of the simulated band structures.

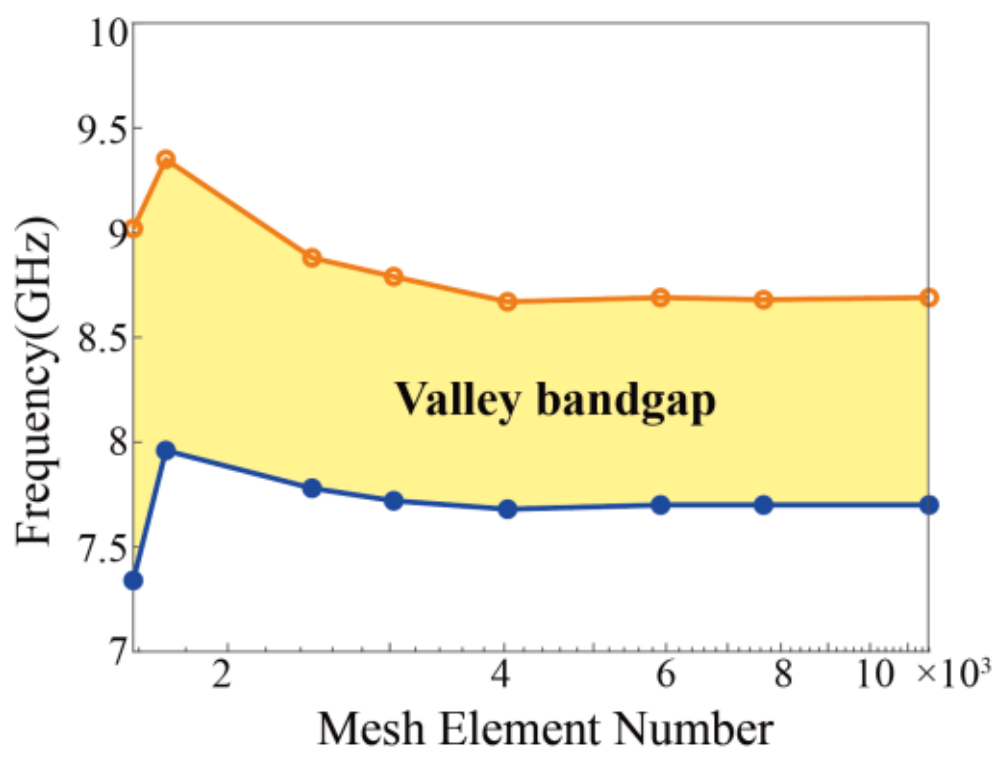

Figure S17. Topological bandgap varies with mesh element number
